# The Rice Endophyte-Derived α-Mannosidase ShAM1 Degrades Host Cell Walls To Activate DAMP-Triggered Immunity against Disease

**DOI:** 10.1128/spectrum.04824-22

**Published:** 2023-05-08

**Authors:** Zhigang Bu, Wei Li, Xiaoli Liu, Ying Liu, Yan Gao, Gang Pei, Rui Zhuo, Kunpeng Cui, Ziwei Qin, Heping Zheng, Jie Wu, Yutong Yang, Pin Su, Meiting Cao, Xianqiu Xiong, Xuanming Liu, Yonghua Zhu

**Affiliations:** a Hunan Province Key Laboratory of Plant Functional Genomics and Developmental Regulation, College of Biology, Hunan University, Changsha, People’s Republic of China; b Key Laboratory of Modern Research of TCM, Education Department of Hunan Province Hunan, University of Chinese Medicine, Changsha, People’s Republic of China; c Bioinformatics Center, College of Biology, Hunan University, Changsha, People’s Republic of China; d Hunan Academy of Agricultural Sciences, Hunan Plant Protection Institute, Changsha, People’s Republic of China; Connecticut Agricultural Experiment Station

**Keywords:** α-mannosidase, plant immunity, disease resistance, damage-associated molecular pattern (DAMP), endophytic *Streptomyces*

## Abstract

Endophytes play an important role in shaping plant growth and immunity. However, the mechanisms for endophyte-induced disease resistance in host plants remain unclear. Here, we screened and isolated the immunity inducer ShAM1 from the endophyte Streptomyces hygroscopicus OsiSh-2, which strongly antagonizes the pathogen Magnaporthe oryzae. Recombinant ShAM1 can trigger rice immune responses and induce hypersensitive responses in various plant species. After infection with M. oryzae, blast resistance was dramatically improved in ShAM1-inoculated rice. In addition, the enhanced disease resistance by ShAM1 was found to occur through a priming strategy and was mainly regulated through the jasmonic acid-ethylene (JA/ET)-dependent signaling pathway. ShAM1 was identified as a novel α-mannosidase, and its induction of immunity is dependent on its enzyme activity. When we incubated ShAM1 with isolated rice cell walls, the release of oligosaccharides was observed. Notably, extracts from the ShAM1-digested cell wall can enhance the disease resistance of the host rice. These results indicated that ShAM1 triggered immune defense against pathogens by damage-associated molecular pattern (DAMP)-related mechanisms. Our work provides a representative example of endophyte-mediated modulation of disease resistance in host plants. The effects of ShAM1 indicate the promise of using active components from endophytes as plant defense elicitors for the management of plant disease.

**IMPORTANCE** The specific biological niche inside host plants allows endophytes to regulate plant disease resistance effectively. However, there have been few reports on the role of active metabolites from endophytes in inducing host disease resistance. In this study, we demonstrated that an identified α-mannosidase protein, ShAM1, secreted by the endophyte *S. hygroscopicus* OsiSh-2 could activate typical plant immunity responses and induce a timely and cost-efficient priming defense against the pathogen M. oryzae in rice. Importantly, we revealed that ShAM1 enhanced plant disease resistance through its hydrolytic enzyme (HE) activity to digest the rice cell wall and release damage-associated molecular patterns. Taken together, these findings provide an example of the interaction mode of endophyte-plant symbionts and suggest that HEs derived from endophytes can be used as environmentally friendly and safe prevention agent for plant disease control.

## INTRODUCTION

Rice blast caused by the foliar fungal pathogen Magnaporthe oryzae is one of the most widespread and destructive plant diseases (1). Currently, there are no effective solutions to eradicate this disease, and indiscriminate use of chemical control agents may lead to the development of pathogen resistance and possible environmental and health hazards (2). The biological control of rice blast has thus received considerable attention due to its high and specific activity against target pathogens, cost-effectiveness, and good adaptability to the environment (3, 4). In recent years, various microorganisms, including *Streptomyces*, *Bacillus*, Pseudomonas, and *Trichoderma* species, have been reported to suppress rice blasts by versatile mechanisms. In addition to directly suppressing the growth of pathogens by secreting antibiotic substances, competing for nutrient space, or degrading virulence factors (5, 6), antagonist microbes can enhance plant resistance against pathogens by activating the plant immune response, named induced systemic resistance (ISR) (7). For example, cyclic lipopeptides produced by endophytic Pseudomonas strains were recently shown to trigger ISR and enhance resistance to M. oryzae on rice (8). The active volatile compound methyl benzoate (MeBA) from *Ampelomyces* sp. and *Cladosporium* sp. was found to be mainly involved in eliciting ISR in Arabidopsis thaliana against the pathogen Pseudomonas syringae pv. tomato DC3000 (9).

The above plant-microbe interactions involve plant immunity systems, wherein multiple processes can sense and recognize exogenous microorganisms and subsequently trigger defense mechanisms (5). The first layer of immune defense, known as pattern-triggered immunity (PTI), is initiated upon the perception of microbe- or pathogen-associated molecular patterns (MAMPs or PAMPs, respectively), such as bacterial lipopolysaccharides and fungal chitin (10–12). As a countermeasure, some pathogens can overcome the first layer by secreting virulence effectors. In turn, plants have developed a second immunity layer, known as effector-triggered immunity (ETI), which involves resistance proteins that recognize specific pathogen effectors to activate their defense responses (13). Although PTI and ETI detect different types of molecules, they seem to use a similar signaling network and eventually converge into many similar downstream responses (14, 15), such as reactive oxygen species (ROS) bursts, callose deposition, the expression of defense-responsive genes, and activation of phytohormone signaling—i.e., salicylic acid (SA) and the jasmonic acid-ethylene (JA/ET) pathway (16, 17). In particular, the hypersensitive response (HR) is a typical immune response in plants triggered by invading microbes (18). HR is characterized by the presence of dead brown cells, which form visible lesions when enough cells die (19). HR is often associated with ETI, but recent studies have revealed that it is also related to PTI (17). As the HR phenomenon is evident in plants, most immunity elicitors are usually identified by injecting them into the leaves of tobacco plants to observe if HR occurs, such as ethylene-inducing xylanase (EIX) from Trichoderma viride and glycoside hydrolase (XEG1) from Phytophthora sojae (20, 21).

Another interesting immune response involves plant cell-wall-derived fragments. To colonize plants, microbes might damage the plant’s cell walls. Under this condition, the components of plant cell wall polysaccharides, including cellulose, hemicellulose, and pectin, are released into the extracellular space and function as endogenous plant-derived signaling molecules. In turn, damage-associated molecular pattern (DAMP)-triggered immunity (DTI) can also elicit a broad range of defense responses in several plant species. As DAMPs are endogenously produced, they are also referred to as MAMPs. There are various molecules referred to as DAMPs (22), such as pectin-derived oligogalacturonides (OGs), oligosaccharides, and cellooligomers (23–25). Moreover, the secreted microbial enzymes, including pectinases, polygalacturonases, glucanases, cellulases, xyloglucanases, and mannosidase, are thus considered to participate in degradation to release plant cell wall fragments as DAMPs (21, 26). For example, pathogen-derived pectinases and endogenous polygalacturonase liberate oligogalacturonides that can activate plant innate immunity by functioning as DAMPs and enhance resistance to the pathogens Botrytis cinerea and P. syringae pv. tomato DC3000 (27). Similarly, cutinase VdCUT11 from Verticillium dahliae induced plant immunity by degrading plant cell wall polymers to release DAMPs (28). However, these kinds of microbial hydrolytic enzymes that play roles in plant’ innate immune systems remain mostly undiscovered.

In our previous study, we found that the rice endophyte Streptomyces hygroscopicus OsiSh-2 and its cell-free culture filtrate (CFC) had strong antagonistic activity against M. oryzae via the action of nigericin and siderophores (3, 29). OsiSh-2 could regulate thiamine biosynthesis and thus assist in blast resistance in the OsiSh-2-rice symbiont (30). Specifically, OsiSh-2 also has ISR activity in rice for resisting M. oryzae, including induction of a priming response (31). Priming is an important strategy that allows hosts to defend against pathogen attacks in a timely and cost-efficient manner because plants in the “priming state” exhibit faster and stronger activation of specific defense responses after pathogen infection than plants that sustain a full-scale defense response (32, 33). As a consequence, the saved energy can drive the growth of the host. By these mechanisms, OsiSh-2 can improve host resistance to rice blast while still sustaining a high yield. However, which active compound produced by OsiSh-2 is involved in ISR and the corresponding molecular mechanism are still not entirely clear.

In this study, we screened, isolated, and obtained the ShAM1 protein from the CFC of OsiSh-2 by HR analysis. This protein was identified as a novel α-mannosidase belonging to the glycosyl hydrolase family 92 (GH92). Purified recombinant ShAM1 can activate typical plant defense responses such as H_2_O_2_ accumulation, callose deposition, amplification of phosphorylated mitogen-activated protein kinase (MAPK) cascade signals, activation of genes related to the hormone signaling pathway, and HR in rice. After infection with M. oryzae, ShAM1 pretreatment could induce a priming response, trigger the expression of JA/ET biosynthesis-related genes, and increase resistance to M. oryzae in rice. As a glycosyl hydrolase, when ShAM1 was coincubated with isolated rice cell walls, the release of oligosaccharides and monosaccharides was observed. Importantly, the extract from the ShAM1-digested cell wall could activate the immune response and enhance the disease resistance of the host rice. Thus, ShAM1 degrades the cell wall to release oligosaccharides and monosaccharides, which might serve as DAMPs to activate host immunity and trigger defense against M. oryzae.

## RESULTS

### ShAM1 from *S. hygroscopicus* OsiSh-2 is an HR-inducing protein.

**FIG 1:**
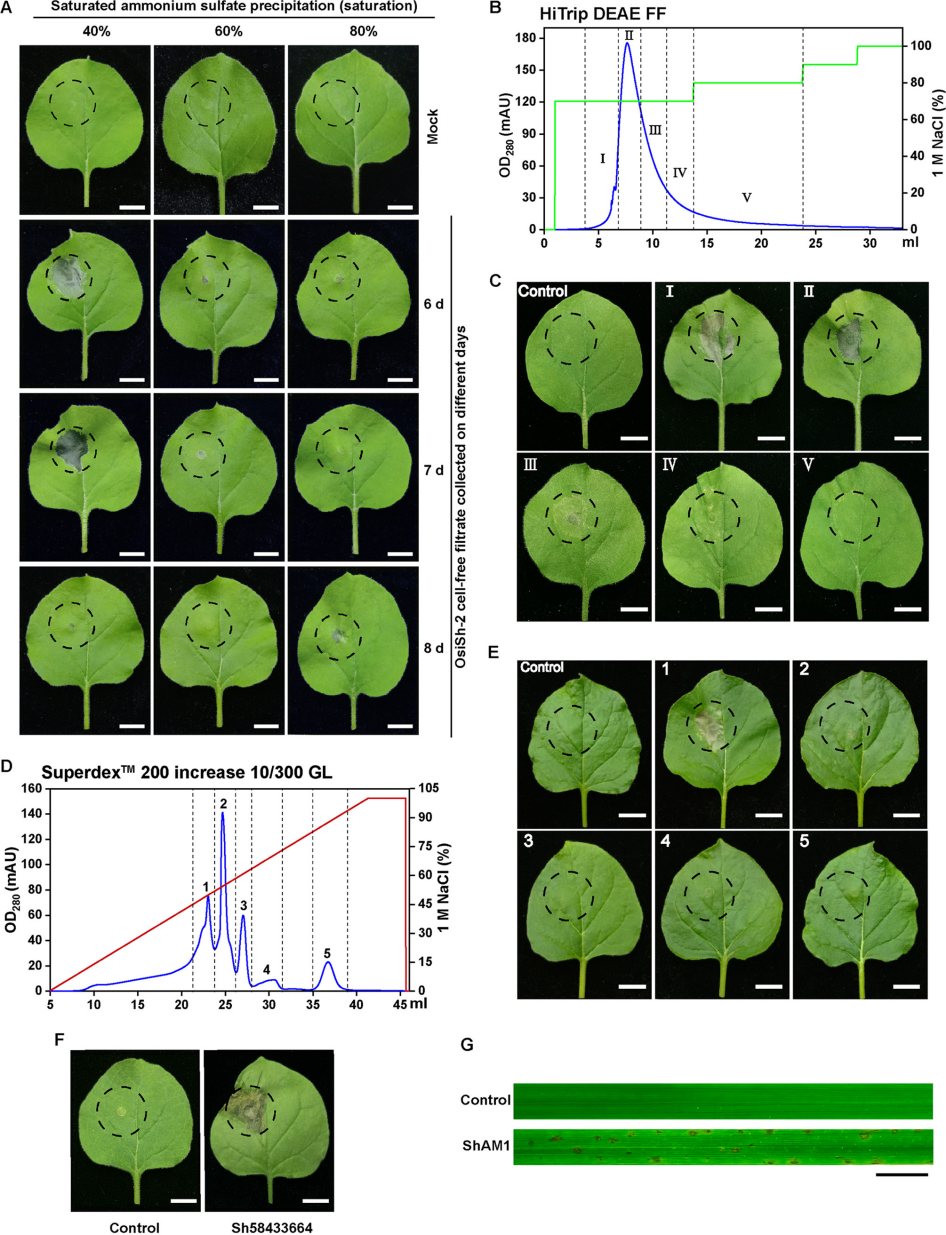
ShAM1 purified from *S. hygroscopicus* OsiSh-2 culture supernatants induces HR in tobacco and rice. (A) Protein precipitation from the 7-day culture using 40% ammonium sulfate caused the strongest HR on tobacco leaves. Buffer (the buffer was treated in the same way as the protein extract) was used as a mock control. Images were taken 48 h after injection. Scale bars, 1 cm. (B) Fractionation of a 40% ammonium sulfate precipitate by column chromatography on the DEAE FF column yielded five fractions (I to V). Blue curve, protein concentration; green curve, salt concentration. (C) The tobacco leaves were infiltrated with fractions from the DEAE FF column or buffer as a control. (The buffer was the International Streptomyces Program 2 liquid medium, and “purified” in the same way as the secreted proteins.) The experiment was repeated three times with similar results. Scale bars, 1 cm. (D) The above fractions (parts I and II were mixed) that could induce HR in tobacco were further separated using gel permeation chromatography (Superdex 200 Increase 10/300 GL), yielding five main peaks (1 to 5). Blue curve, protein concentration; red curve, salt concentration. (E) The concentrated mixture of parts I and II was passed through a gel permeation chromatography column (Superdex 200 Increase 10/300) and yielded five main peaks. Proteins corresponding to peak 1 could induce HR on tobacco leaves. Buffer was taken as a control and “purified” in the same way as the secreted proteins. Images were taken 48 h after injection. The experiment was repeated three times with similar results. Scale bars, 1 cm. (F) The leaves of 4-week-old tobacco plants were inoculated with *Agrobacterium* strains carrying the indicated gene in the vector pCAMBIA-1300-FLAG. The vector pCAMBIA-1300-FLAG was used as a control. The gene *Sh58433664* was designated *ShAM1*. Images were taken 5 days after inoculation. Scale bars, 1 cm. (G) The leaves of 2-week-old rice seedlings were treated with recombinant ShAM1 (3 μM). The vector pCold TF was used as a control. Images were taken 48 h after inoculation. Scale bars, 1 cm.

To identify a protein with immunity-inducing activity from *S. hygroscopicus* OsiSh-2, a systematic purification approach was adopted. In every purification step, each fraction was infiltrated into tobacco leaves to monitor HR. Initially, crude proteins from different days of CFC of OsiSh-2 were precipitated with 40, 60, and 80% saturated ammonium sulfate (SAS) (see Fig. S1 in the supplemental material). As shown in Fig. 1A, protein precipitation from the 7-day culture using 40% SAS caused the strongest HR on tobacco leaves and was thus fractionated by column chromatography on an anion-exchange DEAE Fast Flow (FF) device. Five fractions (I to V) were separately collected (Fig. 1B), and the fractions of parts I and II were selected for further purification due to the presence of HR activity (Fig. 1C). A concentrated mixture of parts I and II was passed through a Superdex 200 Increase 10/300 column and showed 5 distinct prominent peaks (Fig. 1D). Then, the protein samples in peaks 1 and 4, which presented high and no HR activity (Fig. 1E), respectively, were subjected to nano-liquid chromatography-tandem mass spectrometry (NanoLC-MS/MS). We suspected that the only expressed proteins in peak 1 might contain HR-inducing protein candidates. Based on the results of mass spectrometry and a search against the OsiSh-2-specific genome databases using the Mascot tool, 78 differentially abundant proteins between peaks 1 and 4 were found. Gene Ontology (GO) annotation grouped these proteins into two categories, biological processes and molecular functions (Fig. S2), which demonstrated that a large proportion of these proteins were related to carbohydrate metabolic progress and hydrolase activities. In addition, genome functional annotation of OsiSh-2 indicated that among these 78 proteins, 15 were hydrolases, including 8 glycoside hydrolases (see Table S1 in the supplemental material).

To determine whether these 15 hydrolases were responsible for the development of HR, we used Agrobacterium tumefaciens-mediated transformation (agroinfiltration) to transiently express the 15 corresponding coding genes in tobacco leaves (Fig. S3). As shown in Fig. 1F, transient expression of the gene *Sh58433664* (designated *ShAM1*) triggered HR in tobacco 7 days after infiltration. We further heterologously expressed the ShAM1 protein in Escherichia coli BL21(DE3), and the purified recombinant protein induced a strong HR in rice leaves after 48 h of treatment (Fig. 1G; Fig. S5E). In addition, gradual dilution (from 30 nM to 3 μM) of ShAM1 decreased the development of HR, and ShAM1 induced HR at concentrations as low as 150 nM (Fig. S4). To examine the host specificity of ShAM1, we injected ShAM1 (3 μM) into the leaves of various plant species. The results revealed that ShAM1 could induce HR in *Arabidopsis*, pepper, cucumber, tomato, and tobacco (Fig. S5). All of these data indicated that ShAM1 is an HR-inducing protein secreted by OsiSh-2.

### ShAM1 activates immune responses in rice.

**FIG 2:**
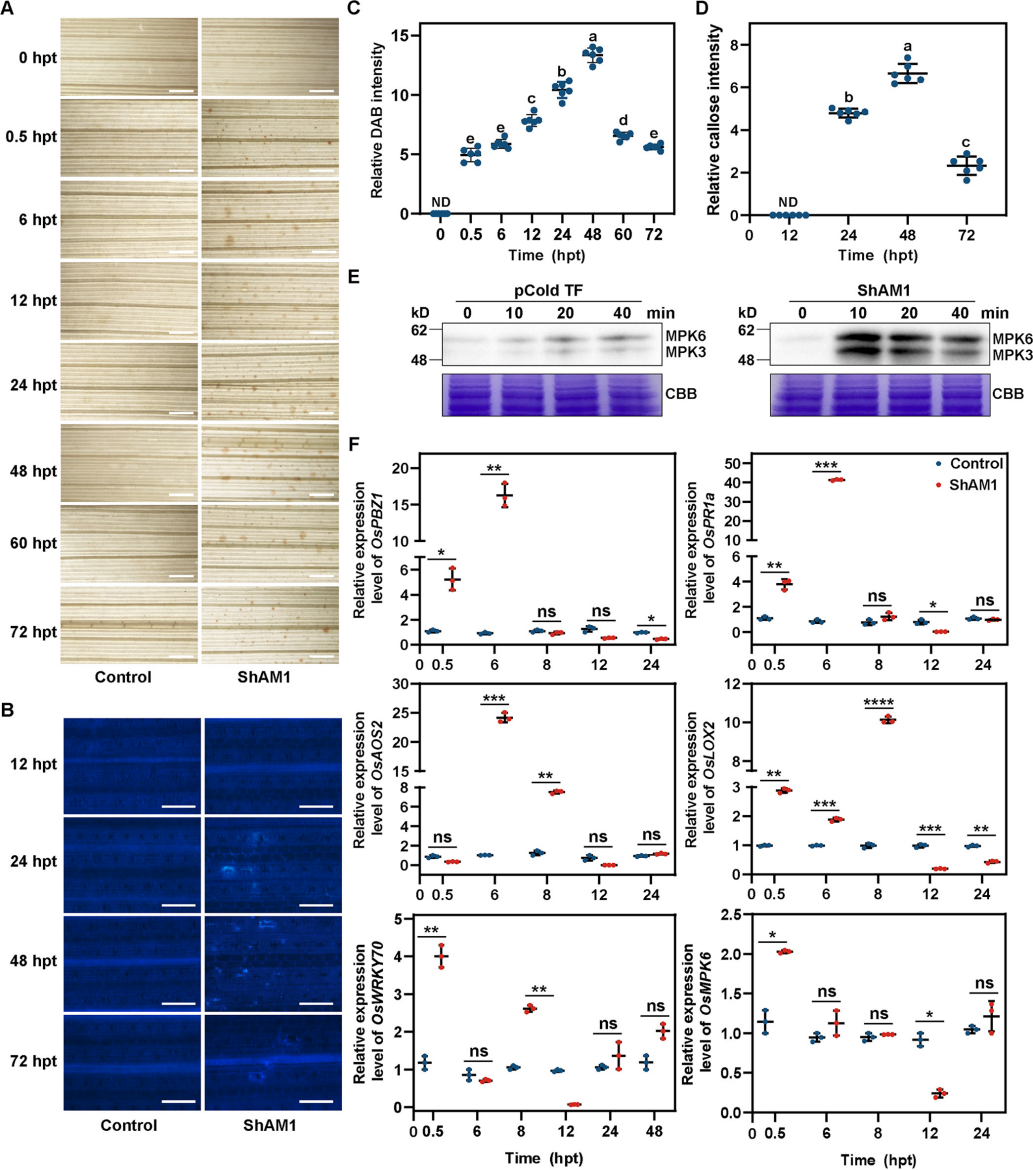
ShAM1 activated immune responses in rice. (A) ShAM1 induced H_2_O_2_ accumulation in rice. The leaves of 2-week-old rice seedlings were treated with purified recombinant ShAM1 (3 μM) and then stained using 3,3′-diaminobenzidine (DAB). Images were taken at different time points after treatment. Scale bars, 1 mm. (B) ShAM1 induced callose deposition. The leaves of rice were treated with ShAM1 as described for panel A and then stained using aniline blue. Images were taken at different time points after treatment. Scale bars, 100 μm. (C) The abundance of H_2_O_2_ accumulation is represented by the percentage of relative DAB intensities, which were calculated by Photoshop CS6 and ImageJ based on counting the numbers of DAB-stained pixels. The data shown indicate the means ± standard deviations (SDs). Bars with different letters are significantly different (ANOVA; *P* < 0.05) according to Duncan’s multiple-range test. (D) The abundances of callose intensities are represented by the percentages of relative callose intensities, which were calculated by using Photoshop CS6 and ImageJ based on counting the numbers of callose staining pixels. The data shown indicate the means ± SDs. The experiment was repeated three times with similar results. ND, not detectable. Bars with different letters are significantly different (ANOVA; *P* < 0.05) according to Duncan’s multiple-range test. (E) ShAM1 protein can activate MAPK signaling in rice. ShAM1 protein (3 μM) was incubated with rice suspension cells. pCold TF protein (3 μM) was used as the negative control. Activated MAPKs were determined with the phospho-p38 MAPK antibody at the indicated times. The corresponding bands are represented for the phosphorylation of MPK3 and MPK6. Coomassie brilliant blue (CBB) staining of Rubisco was used to ensure equal loading in each lane. The experiment was repeated three times with similar results. (F) ShAM1 upregulated the expression of immune-related genes. ShAM1-treated rice was the same as in panel A, and the samples were collected for RT-qPCR at the indicated time points. The data shown indicate the means ± SDs (*n* = 3; *n* refers to biological replicates). ns, not significant. Bars with different asterisks are significantly different, as determined by the Tukey-Kramer test (*, *P* < 0.05; **, *P* < 0.01; ***, *P* < 0.001; ****, *P* < 0.0001).

HR is usually associated with the activation of plant innate immunity. Thus, some key components of the plant immune response were determined. With regard to ROS production, rapid and slight accumulation of H_2_O_2_ at 0.5 h posttreatment (hpt) in the veins of rice was observed after being spray purified with recombinant ShAM1. The H_2_O_2_ content peaked at 48 hpt and then decreased to a low level. However, no H_2_O_2_ accumulation was detected in vector pCold TF-treated (control) rice (Fig. 2A and C). As a result, the downstream immune response of the H_2_O_2_ messenger, the deposition of callose, which works as an effective barrier in the plant cell wall for blocking subsequent pathogen invasion, was induced (34). At 24 hpt, callose deposition around the stomata was observed in ShAM1-treated rice leaves, whereas no callose deposition was found in control rice leaves (Fig. 2B, D).

To further verify the above observations, we tested ShAM1-induced activation of phosphorylated MAPK proteins and immune-responsive gene expression. When ShAM1 was exogenously applied to rice cells, we found that the MAPK pathway was significantly activated, while the control treatment did not activate this pathway (Fig. 2E). The expression of genes associated with the SA signaling pathways (*OsPBZ1* and *OsPR1a*), JA/ET signaling pathways (*OsAOS2* and *OsLOX2*), and cross talk between the SA and JA/ET signaling pathways (*OsWRKY70* and *OsMPK6*) was then examined. Similar to ShAM1-induced MAPK activation, all of the genes were significantly upregulated in the early stage of treatment. For example, the expression of *OsPR1a* and *OsAOS2* increased as much as 45.9- or 22.9-fold, respectively, at 6 hpt (Fig. 2F). These results confirmed that ShAM1 effectively activated the rice immune response.

### ShAM1 pretreatment induced rice defense priming and enhanced resistance to M. oryzae in rice.

**FIG 3:**
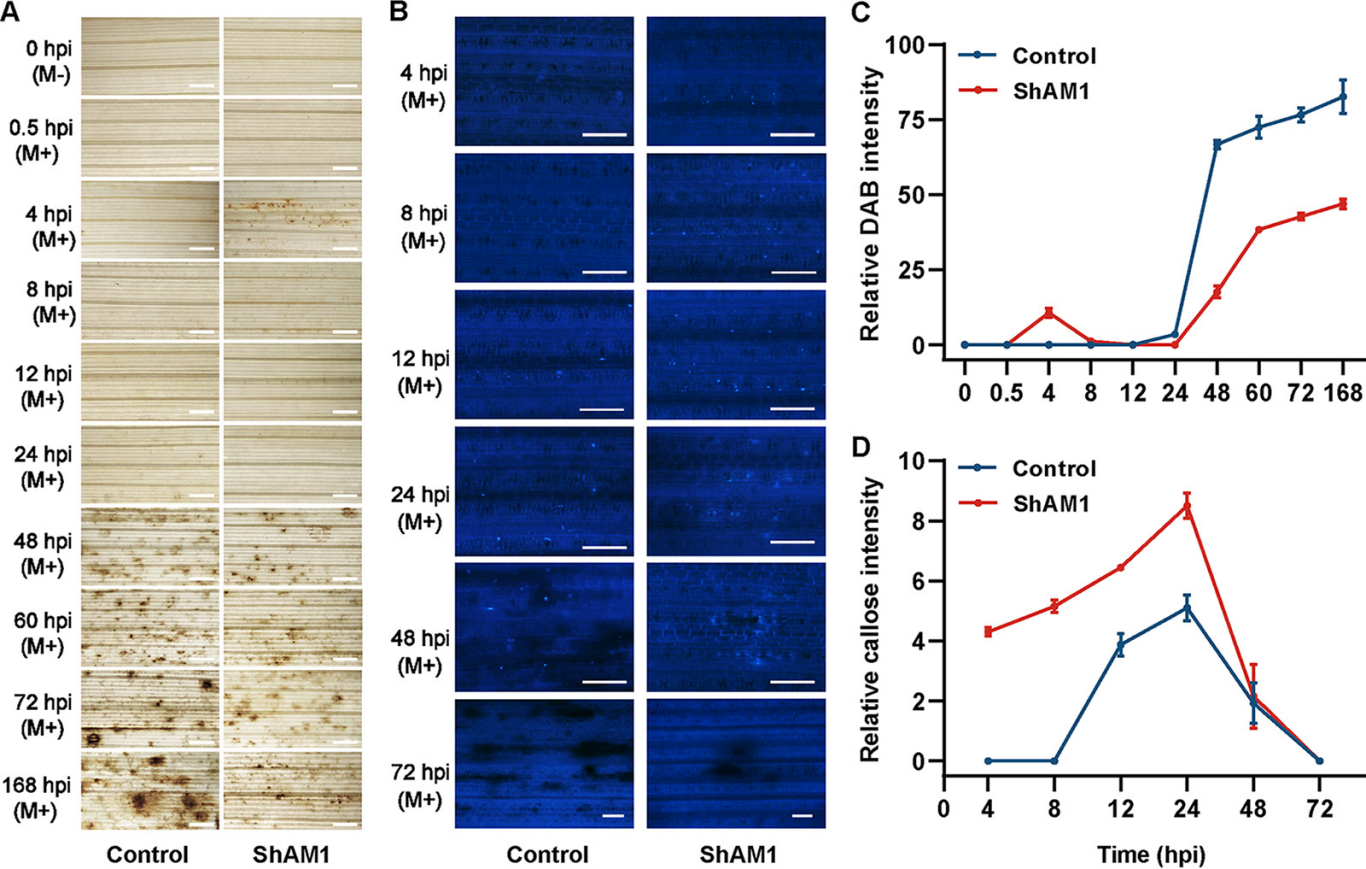
ShAM1 triggers defense priming in rice leaves. (A) H_2_O_2_ accumulation is induced in ShAM1-pretreated rice leaves after M. oryzae infection. Stereomicroscopic observation of H_2_O_2_ accumulation (as indicated by DAB staining) in ShAM1-pretreated rice at different times postinfection (hpi) by M. oryzae was performed. pCold TF-pretreated rice was used as a control. Scale bars, 1 mm. (B) Callose deposition is induced in ShAM1-pretreated rice leaves after M. oryzae infection. Fluorescence microscopy observation of callose deposition in the same samples at the indicated times postinfection (hpi) by M. oryzae and then stained using aniline blue was performed. pCold TF-pretreated rice was used as a control. Scale bars, 100 μm. (C) The abundance of H_2_O_2_ accumulation is represented as the percentage of relative DAB intensities in ShAM1-pretreated rice leaves at the indicated hpi by M. oryzae. The data shown indicate the means ± SDs. (D) The abundances of callose intensities are represented by the percentage of relative callose intensities. The data shown indicate the means ± SDs.

Our previous studies indicated that OsiSh-2 could induce a priming response, an adaptive strategy to improve the defensive capacity of host rice to the pathogen. As an immunity-related active protein of OsiSh-2, ShAM1 is likely to play a role in keeping the host rice in the priming state. When M. oryzae was infected, a significant ROS burst was observed in both ShAM1-pretreated and pCold TF-pretreated (control) rice, but this response in the former was faster. H_2_O_2_ accumulated in the ShAM1-pretreated rice leaves at 4 h postinfection (hpi) by M. oryzae, while the H_2_O_2_ in control rice just began to largely accumulate at 24 hpi and the level further increased thereafter (Fig. 3A and C). Similarly, callose deposition in ShAM1-pretreated rice occurred at least 4 h earlier than that in control rice, and the amount was also dramatically increased in ShAM1-pretreated rice (Fig. 3B and D). These results, especially the callose data, presented a typical defense priming response pattern in ShAM1-pretreated rice, indicating that ShAM1 could induce an immune response in rice by priming.

**FIG 4:**
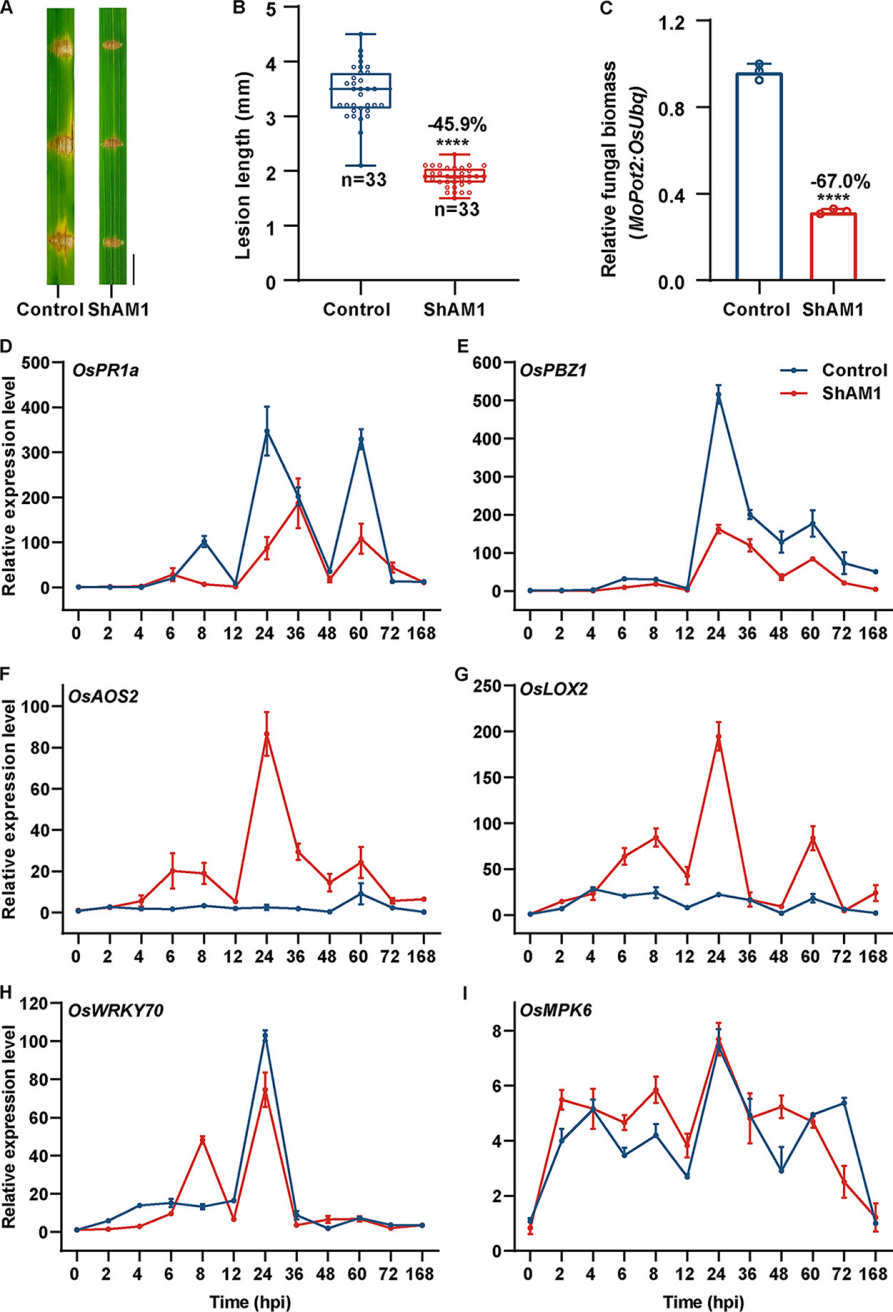
ShAM1 increased blast disease resistance in rice. (A) Images of blast lesions on detached rice leaf segments 5 days after punch inoculation with M. oryzae 70-15 at a concentration of 5 × 10^5^ per mL. Scale bars, 0.5 mm. (B) The lesion length was measured at 5 days postinoculation. Experimental repeats are displayed as box plots with individual data points (*n* = 33). The error bars represent maximum and minimum values. Middle horizontal bars of box plots represent the median, and the bottom and top represent the 25th and 75th percentiles, respectively. Statistical significance (****, *P* < 0.0001) was revealed by Student's *t* test. The percentage changes followed by “+” or “−” above the bars were calculated by using the following formula: % change = [(value of treated rice) − (value of untreated rice)/(value of untreated rice)] × 100%. (C) ShAM1-pretreated rice inhibited the growth of M. oryzae. The relative fungal biomass was determined by qPCR. The relative fungal growth of M. oryzae was calculated using the threshold cycle value (*C_T_*) of *MoPot2* DNA (an inverted repeat transposon of M. oryzae) versus the *C_T_* of *OsUbq* DNA (a rice genomic ubiquitin gene) by DNA-based qRT-PCR at 5 days postinfection by M. oryzae in rice leaves. The data shown indicate the means ± SDs (*n* = 3; *n* refers to biological replicates). Statistical significance (****, *P* < 0.0001) was revealed by Student's *t* test. The percentage changes followed by “+” or “−” above the bars were calculated by using the above formula (D to I) The expression levels of JA/ET pathway marker genes were significantly upregulated in ShAM1-pretreated rice after M. oryzae infection. ShAM1 was inoculated with 2-week-old rice seedlings and then infected with M. oryzae after recovery for 7 days, and samples were collected for qRT-PCR at 5 days postinfection by M. oryzae in rice leaves. The reference gene *OsUbq* (a rice genomic ubiquitin gene) was used for the normalization of all qRT-PCR data. pCold TF-pretreated rice was used as a control. The data shown indicate the means ± SDs (*n* = 3; *n* refers to biological replicates).

We then evaluated the resistance effect of ShAM1 against rice blasts. As shown in Fig. 4A and B, the blast lesions of ShAM1-pretreated rice were significantly smaller than those of control rice, with a 45.9% reduction. Correspondingly, the relative fungal biomass, as indicated by the ratio of the *MoPot2* gene (an inverted repeat transposon of M. oryzae) to the *OsUbq* gene (a rice genomic ubiquitin gene) in ShAM1-pretreated rice, showed a 67.0% reduction compared with that in control rice (Fig. 4C). Interestingly, among the defense-related genes, the marker genes in the SA and JA/ET pathways showed different profiles between ShAM1-pretreated and control rice after M. oryzae infection. The pathogenesis-related genes *OsPR1a* and *OsPBZ1* of the SA signaling pathway were strongly activated by M. oryzae in control rice, but ShAM1 pretreatment led to decreased expression of these two genes (Fig. 4D and E). In contrast, the expression of the JA/ET biosynthesis-related genes *OsAOS2* and *OsLOX*2 was strongly triggered in ShAM1-pretreated rice (Fig. 4F and G), whereas they were not induced by M. oryzae infection in control rice. For *OsWRKY70* and *OsMPK6*, there were no obvious differences between these two groups of rice (Fig. 4H and I). These results indicated that ShAM1 might regulate the immune responses in rice to enhance its resistance to M. oryzae mainly through the JA/ET signaling pathway.

### ShAM1 is a novel α-mannosidase, and its enzymatic activity is related to immune induction.

**FIG 5:**
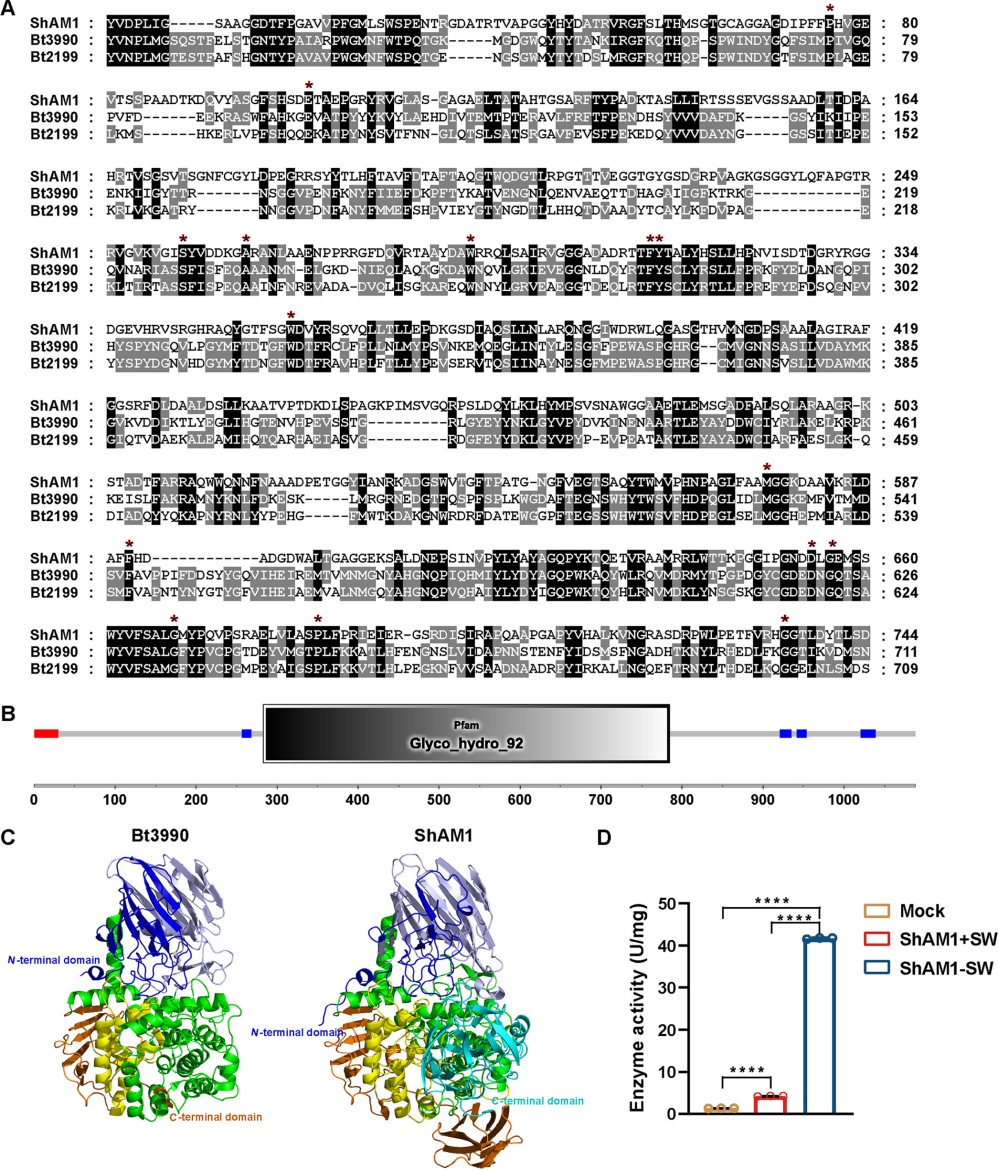
Characterization of recombinant α-mannosidase ShAM1. (A) Conserved amino acid residues of several ShAM1 homolog proteins from B. thetaiotaomicron (Bt3990 and Bt2199). Amino acid sequence similarity analysis of Bt3990 and Bt2199 was performed. Sequences in black borders indicate conserved domains. Residues conserved in all GH92 enzymes are indicated by an asterisk. The sequences were aligned using ClustalW. (B) ShAM1 belongs to the glycosyl hydrolase GH92 family. The conserved ShAM1 domains were predicted using the SMART database. (C) Predicted three-dimensional (3D) protein structure models of ShAM1 and Bt3990 generated by AlphaFold; (D) SW inhibited ShAM1 enzyme activity. ShAM1 was mixed with the indicated amounts of SW (final concentration of 40 μM), and after preincubation for 1 h at 40°C, the reactions were started by the addition of pNP-α-d-man (5 mM). SW, swainsonine. Buffer was used as the mock treatment. The data shown indicate the means ± SDs (*n* = 3; *n* refers to biological replicates). Statistical significance (****, *P* < 0.0001) was revealed by Student's *t* test.

ShAM1 was predicted to be a glycosyl hydrolase with a molecular weight of 113.7 kDa (Table S1). The protein sequences of ShAM1 contained a GH92 domain predicted by the SMART database (Fig. 5B). ShAM1 is categorized as a secreted protein that contains a signal peptide but lacks transmembrane domains. Comparison of the sequence of ShAM1 with those of the reported α-mannosidases showed that ShAM1 shares 31% identity with the α-mannosidase Bt3990 from Bacteroides thetaiotaomicron (35) (Fig. 5A). The predicted three-dimensional (3D) protein structure from AlphaFold also indicated that ShAM1 was highly similar to Bt3990 (Fig. 5C).

In natural environments, α-mannosidase from microbes is often employed in the hydrolyzation of α-mannosidic linkages from both high-mannose-type and plant complex-type N-glycans (36). To identify the enzyme activity of recombinant ShAM1, we tested the activities using different special substrates for different enzymes. That is, 4-nitrophenyl-α-d-mannopyranoside (pNP-α-d-man) was used for α-mannosidase activity, 4-nitrophenyl-β-d-mannopyranoside (pNP-β-d-man) and locust bean gum (LBG) were used for β-mannosidase activity, 4-nitrophenyl-α-d-glucopyranoside and 4-nitrophenyl-β-d-glucopyranoside were used for glucosidase activity, microcrystalline cellulose was used for cellulase activity and xylan was used for xylanase activity. The results showed that ShAM1 only exhibits the expected specificity for pNP-α-d-man (Table S2), indicating that ShAM1 is a novel α-mannosidase rather than β-mannosidase, which is usually found in bacteria, plants, and fungi. To further verify the α-mannosidase activity of ShAM1, swainsonine (SW), an alkaloid that specifically inhibits α-mannosidase, was added (37). As shown in Fig. 5D, the activity of ShAM1 incubated with SW was decreased by 89.9%.

The optimal pH for recombinant ShAM1 activity was 5.5 (Fig. S6A), and the stable pH ranged from 5.5 to 6.5 (Fig. S6B). Moreover, the optimal temperature was approximately ~40°C (Fig. S6C), and 80% relative activity was retained at 45°C (Fig. S6D). Various metal ions showed different effects on the activity of the α-mannosidase ShAM1. As shown in Fig. S6E, only Ca^2+^ significantly promoted the activity of ShAM1 with a 173.63% increase, while other metal ions, such as Zn^2+^, Cu^2+^, and Pb^2+^, strongly inhibited ShAM1 activity.

To determine whether the immune response triggered by ShAM1 is related to its hydrolase activity, high-temperature-treated (preincubated at 100°C for 20 min) or SW inhibitor-pretreated ShAM1 was infiltrated into tobacco leaves to monitor HR activity. As shown in Fig. 7 below, inactivation treatment of ShAM1 could not induce HR in tobacco, indicating that the enzymatic activity of ShAM1 is required for its immune induction.

### ShAM1 hydrolyzes the rice cell wall to produce DAMPs to activate the immune response in rice and enhance rice blast resistance.

**FIG 6:**
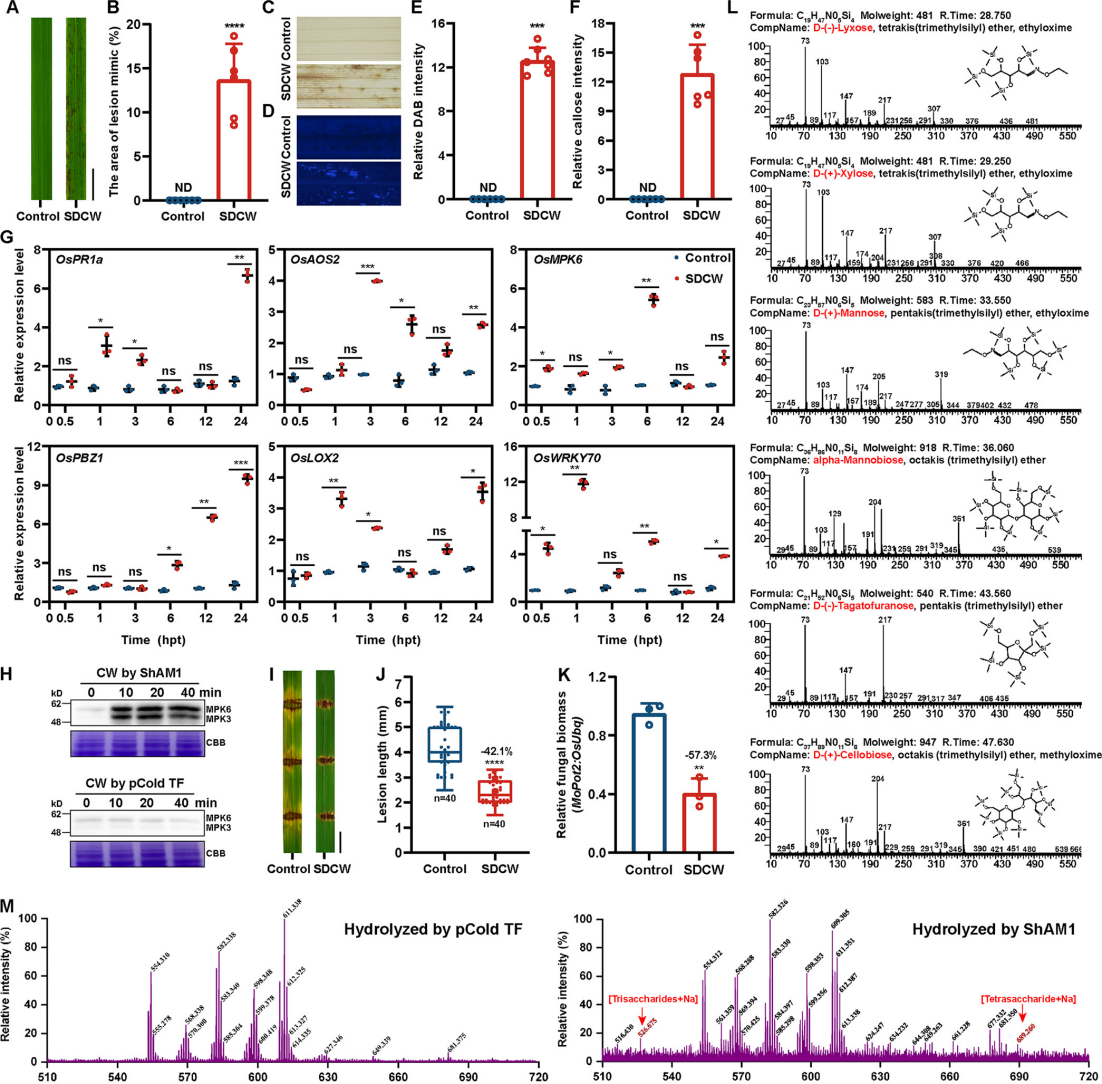
ShAM1 hydrolysis of the rice cell wall activates an immune response in rice. (A) ShAM1-digested cell wall (SDCW) extracts induced HR in rice. The isolated rice cell walls were incubated with 10 μg recombinant ShAM1 or pCold TF. After incubation, samples were boiled for 20 min to denature the protein. The supernatant was used as the elicitor. The leaves of 2-week-old rice seedlings were treated with ShAM1-digested cell wall extracts or the control. pCold TF-digested cell wall extracts were used as controls. (B) Images were taken 48 h after inoculation. Scale bars, 1 cm. The area of the lesion mimic was calculated by ImageJ software. The data shown indicate the means ± SDs. Statistical significance (****, *P* < 0.0001) was revealed by Student's *t* test. (C) SDCW extracts induced H_2_O_2_ accumulation in rice. The leaves of rice were treated with SDCW extracts as described for panel A. Images were taken 48 h after treatment. Scale bars, 1 mm. (D) SDCW extracts induced callose deposition in rice. Images were taken 48 h after treatment. Scale bars, 100 μm. (E) The abundance of H_2_O_2_ accumulation is represented by the percentage of relative DAB intensities. The data shown indicate the means ± SDs. ND, not detectable. Statistical significance (****, *P* < 0.0001) was revealed by Student's *t* test. (F) The abundances of callose intensities are represented by the percentages of relative callose intensities. Statistical significance (****, *P* < 0.0001) was revealed by Student's *t* test. (G) SDCW extracts induced immune-related gene expression. SDCW extract-treated rice was the same as in panel A, and the samples were collected for RT-qPCR at the indicated time points. The data shown indicate the means ± SDs (*n* = 3; *n* refers to biological replicates). ns, not significant. Bars with different asterisks are significantly different, as determined by the Tukey-Kramer test (*, *P* < 0.05; **, *P* < 0.01; ***, *P* < 0.001). (H) ShAM1-hydrolyzed rice cell wall activated MAPK cascades. The rice suspension cells were treated with the ShAM1- or pCold TF-hydrolyzed rice cell wall extracts and were used for MAPK activation assays. As in Fig. 2E, the MAPK level was determined with the phospho-p38 MAPK antibody. The pCold TF-hydrolyzed rice cell wall extract treatment was used as a control. CW, cell wall. The experiment was repeated three times with similar results. (I) SDCW extract-pretreated rice exhibited enhanced disease resistance to M. oryzae. Shown are images of blast lesions on detached rice leaf segments 5 days after punch inoculation with M. oryzae 70-15 at a concentration of 5 × 10^5^ per mL. Scale bars, 0.5 mm. (J) The lesion length was measured at 5 days postinoculation (dpi). Experimental repeats are displayed as box plots with individual data points (*n* = 40). (K) SDCW extract-pretreated rice inhibited the growth of M. oryzae. The data shown indicate the means ± SDs (*n* = 3; *n* refers to biological replicates). Statistical significance (****, *P* < 0.0001) was revealed by Student's *t* test. Data statistics are described in the legend to Fig. 4A to C. (L) The monosaccharides and disaccharides released from ShAM1-hydrolyzed cell walls were detected by GC-MS. The filtered supernatants of ShAM1- and pCold TF-hydrolyzed rice cell walls were used for the analysis. Oligosaccharides and monosaccharides were prepared by sugar oxime trimethylsilylation derivatization, and the mass spectrum was compared with the NIST2014 and NIST2017 spectrum retrieval databases to determine the structures of the compounds. Six carbohydrates were identified. (M) Trisaccharides and tetrasaccharides released by ShAM1 from the rice cell wall. The oligosaccharides of the ShAM1-hydrolyzed cell walls were detected by MALDI-TOF MS. The filtered supernatants of ShAM1-hydrolyzed rice cell walls were used for the analysis, using 2,5-dihydroxybenzoic acid (10 mg/mL in 2:1 acetonitrile-water containing 0.1% TFA) as the matrix. All indicated peaks are single-charged ions of oligosaccharides on the reducing end with Na^+^ using 3-aminoquinoline (3-AMQ) as the ionic liquid matrix.

Generally, protein-induced rice immunity may be related to a PAMP that is directly recognized by a receptor or functions as an enzyme to release cell wall fragments as DAMPs. As ShAM1-induced HR is enzyme dependent, the scenario of rice cell-wall-derived DAMPs seems more likely. Thus, we tested whether ShAM1-digested cell wall (SDCW) extracts could activate the plant immune response. pCold TF-digested cell wall extracts were used as controls. As shown in Fig. 6, strong HR was induced in SDCW extract-treated rice (Fig. 6A and B), and a large amount of H_2_O_2_ and callose accumulation was observed in SDCW extract-treated rice at 48 hpt (Fig. 6C to F), while the control rice showed no immune response. Moreover, SA and JA/ET signaling pathway-related genes were significantly upregulated compared with those in control rice in under early treatment (Fig. 6G). We further demonstrated that the ShAM1 hydrolysis of the cell wall induced strong MAPK activation in rice cells, while the control treatment did not activate this pathway (Fig. 6H). Regarding resistance to rice blast, lesion lengths were reduced by 42.1% in SDCW extract-pretreated rice compared with control rice, which showed typical blast lesions when infected with M. oryzae (Fig. 6I and J). Accordingly, DNA-based quantitative reverse transcription-PCR (qRT-PCR) determination of M. oryzae biomass in the rice leaves revealed that the colonization amount in SDCW extract-pretreated rice was decreased by 57.3% compared with that in control rice (Fig. 6K). In addition, we also degraded tobacco cell walls with ShAM1 and then injected or sprayed the tobacco with the extracts of SDCW. As shown in Fig. S8, SDCW could induce HR in tobacco, indicating immune activation by ShAM1 in other species via a model similar to that in rice.

To further confirm that SDCW extracts could serve as DAMPs, we analyzed the extract from the ShAM1-digested cell wall by gas chromatography-mass spectrometry (GC-MS), and four monosaccharides [d-(−)-lyxose, d-(+)-xylose, d-(+)-mannose, and d-(−)-tagatofuranose] and two oligosaccharides [α-mannobiose and d-(+)-cellobiose] were identified (Fig. 6L). In addition, matrix-assisted laser desorption ionization–time of flight mass spectrometry (MALDI-TOF MS) analysis revealed that a trisaccharide and a tetrasaccharide were also released from the ShAM1-digested rice cell wall (Fig. 6M). The oligomers of homogalacturonan, cellulose, mixed-linked glucans, arabinoxylan, and xyloglucan have been reported as DAMPs involved in plant immunity (24, 38–41). Collectively, these results provide evidence for the above hypothesis that ShAM1 activates the immune response and enhances rice blast resistance by digestion of rice cell-wall-derived DAMPs.

## DISCUSSION

**FIG 7:**
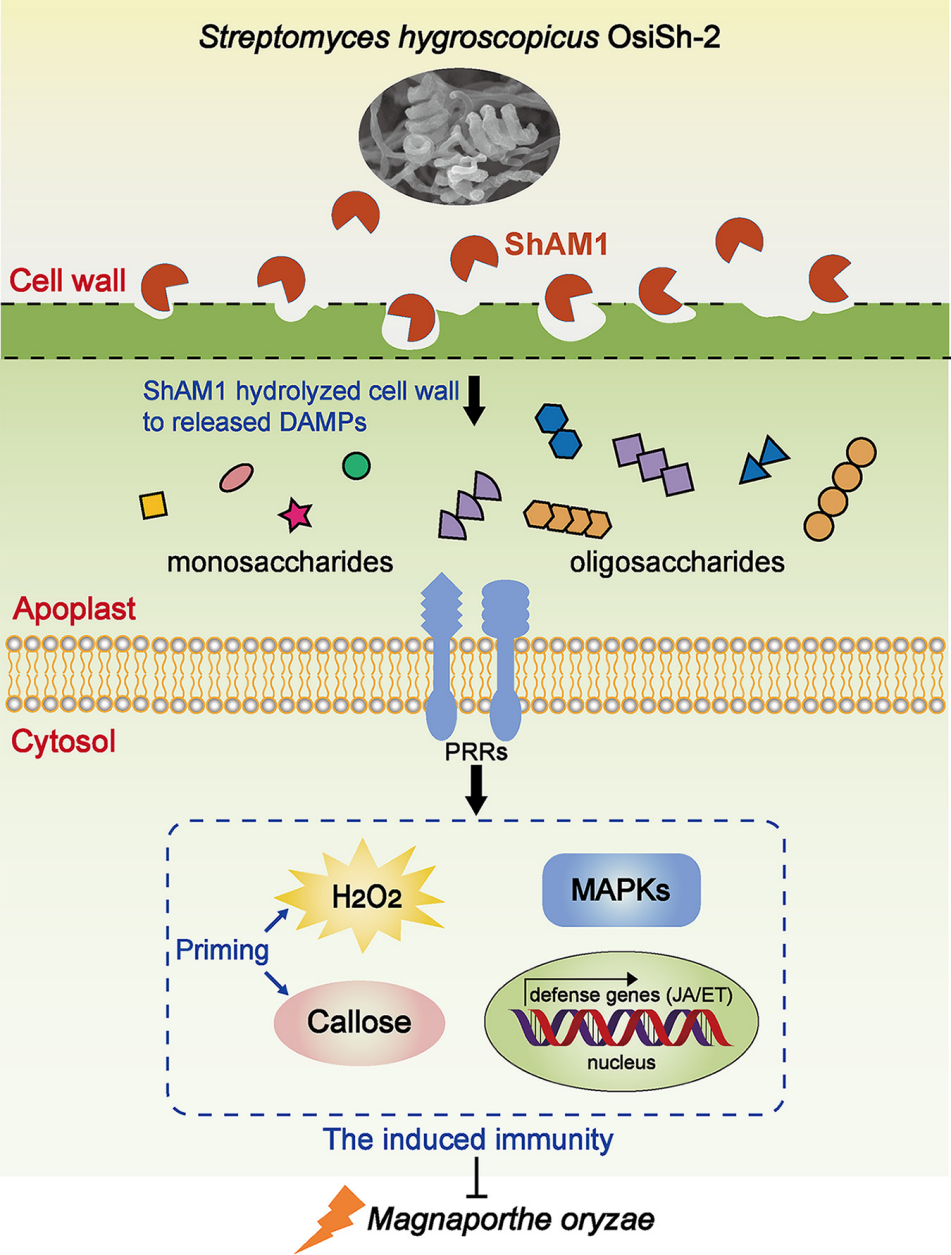
Model of the function of ShAM1 in host plant immunity. The α-mannosidase ShAM1 secreted by endophytic *S. hygroscopicus* OsiSh-2 degrades the cell wall to release oligosaccharides and monosaccharides, which might serve as DAMPs to activate the immune response. A priming state and JA/ET-dependent signaling pathway in the host rice were then activated. Consequently, ShAM1-treated rice showed enhanced disease resistance to M. oryzae.

Currently, many reports have focused on the application of endophytes with the capability to produce valuable bioactive molecules as biocontrol agents, as the specific biological niche inside host plants makes endophytes efficient in disease resistance for the endophyte-plant symbiont (42). However, as a kind of microbe with the capability of producing a broad spectrum of secondary metabolites with diverse biological activities, actinomycetes, and their active metabolites are scarcely reported to play a role in inducing host disease resistance, let alone endophytic actinomycetes. In this study, we isolated a new participant in the plant immune response, ShAM1, from the rice endophytic actinomycete OsiSh-2. ShAM1 was identified as a novel α-mannosidase (Fig. 5; see Table S2 in the supplemental material) and further heterologously expressed in E. coli BL21(DE3). Recombinant ShAM1 could trigger a typical HR in rice, tobacco, *Arabidopsis*, pepper, cucumber, and tomato, indicating its broad spectrum of immunity-inducing activities. Spraying ShAM1 led to a cascade of immune responses in rice, including the production of H_2_O_2_, deposition of callose, amplification of phosphorylated MAPK cascade signals, and expression of several immune-responsive genes (Fig. 2). Consequently, when infected by M. oryzae, ShAM1-pretreated rice showed enhanced resistance to the pathogen (Fig. 3 and 4). Interestingly, ShAM1 is a glycosyl hydrolase, and its induced immunity is enzyme activity dependent. Thus, we proposed that ShAM1 activates immunity and enhances disease resistance in rice by degrading rice cell walls to release DAMPs based on the following elaboration (Fig. 7).

As the first physical barrier to defend against microbial invasion, the plant cell wall is involved in sensing external stresses and transferring the corresponding signal to stimulate defense responses, known as PAMP-triggered immunity. The plant cell wall has a dynamic and highly regulated structure consisting mainly of carbohydrate-based polymers, including cellulose, hemicelluloses, pectin polysaccharides, and minor proportions of mannan and glucomannan (43). Many microorganisms have evolved enzymatic systems capable of degrading plant-related macromolecules (44). Thus, many glycosyl hydrolases (GHs) secreted by microbes can be directly recognized by receptors in plant cell walls. For example, the GH12 protein XEG1 from the pathogen *P. sojae* (21, 45) and two GH12 proteins, VdEG1 and VdEG3, with cellulase activity from the pathogen V. dahliae (28) are recognized via receptor-like proteins in host plants and activate the defense response. These triggered immunizations are independent of GH enzymatic activities.

It should be noted that some GHs have been identified to induce plant immune responses via the DAMP mechanism (46), a currently reported important model of plant immunity (22). These GHs break down cell wall polymers, and the products act as DAMPs to regulate immune responses (47). That is, these triggered immunizations are dependent on GH enzymatic activities. For example, a GH17 enzyme from the pathogen Cladosporium fulvum was proposed to disrupt tomato cell walls and trigger host immune responses (48). The pathogen M. oryzae secretes two GH12 endoglucanases, MoCel12A and MoCel12B, to degrade rice cell walls and release trisaccharide and tetrasaccharide to activate immune responses and increase blast disease resistance in rice (16). A GH5 protein from the bacterium Bacillus pumilus GBSW19, BpMan5, has β-mannosidase activity to degrade polysaccharides to release oligosaccharides that serve as DAMPs to activate the immune response and protect rice and tobacco against the pathogens Xanthomonas oryzae and Phytophthora nicotianae (26, 49). Here, we infiltrated tobacco leaves with high-temperature-inactivated ShAM1 alone or with an SW inhibitor (see Fig. S7 in the supplemental material) and found no HR induction in tobacco. suggesting that immunity activation depends on enzyme activity. In addition, Y. Gui has reported a secreted protein cutinase, VdCUT11, from the pathogen V. dahliae could activate plant immunity by secreting the protein in plant apoplast to degrade plant cell wall polymers and release DAMPs. In this study, the signal peptide of the ShAM1 protein was predicted by the Conserved Domain Database and SignalP 5.0 server, respectively (Fig. 5B). It was a secreted protein with a predicted N-terminal signal peptide (amino acids 1 to 29). Transient expression of the ShAM1 triggered HR in tobacco 5 to 7 days after treatment (Fig. 1F), while spraying it onto the tobacco leaves with the extracts of ShAM1-digested cell walls could trigger HR in 2 to 3 days (Fig. S8). Transiently expressed ShAM1 protein took a longer time in inducing HR in tobacco than ShAM1-digested cell wall products, which may be due to the process required for ShAM1 to be secreted into the apoplast, where ShAM1 degrades tobacco cell walls and release of DAMPs. However, we have not directly tested whether ShAM1 could be secreted into the apoplast to act on tobacco. It is possible that ShAM1 may have some alternative functions in plant cells leading to HR, which deserves further research.

MALDI-TOF MS and GC-MS analysis revealed that ShAM1 can degrade the rice cell wall to release oligosaccharides (Fig. 6L and M). Mannans are intimately associated with cellulose microfibrils (50). Therefore, it is reasonable that although ShAM1 is an α-mannosidase that is highly specific for α-mannose residues, it can release oligosaccharides by degrading plant polysaccharides. To date, research on α-mannosidases has mainly focused on the diseases caused by the abnormal function of α-mannosidases and the effects of α-mannosidases on fruit ripening and storage (51, 52). This is the first report about α-mannosidases serving as cell-wall-degrading enzymes.

As a novel α-mannosidase belonging to the GH92 family. ShAM1 shares only 31% amino acid sequence identity with the B. thetaiotaomicron α-mannosidase Bt3990, and its 3D structure is highly similar to that of Bt3990 (35) (Fig. 5A to C). In addition, the optimal pH of 5.5 for recombinant ShAM1 activity against pNP-α-d-man was similar to the optimal pH (slightly acidic) reported for many reported α-mannosidases (53, 54). However, ShAM1 has specific enzymatic properties. For example, the enzyme activity of ShAM1 is independent of the presence of Ca^2+^ and can only be enhanced in the presence of Ca^2+^ (Fig. S6E), while by far reported α-mannosidases are dependent on Ca^2+^ for their enzymatic activity. Future studies will focus on biocontrol agent preparation to facilitate widespread application.

Oligosaccharides are typical DAMPs that have been studied and applied as a plant defense elicitor for several years. The cellotriose derived from the endophytic fungus Piriformospora indica induces the elevation of calcium, production of ROS, changes in membrane potential, and the expression of defense-related genes in *Arabidopsis* (55). Pretreatment of *Arabidopsis* with cellobiose increases resistance to P. syringae pv. tomato DC3000 via the induction of a series of immune responses, including fast and short-lived intracellular calcium elevation and activation of kinase (MAPK) cascades, but with no ROS production or callose deposition (24). Pretreatment of *Arabidopsis* with pentasaccharide 3^3^-α-l-arabinofuranosyl-xylotetraose (XA3XX) from arabinoxylan can trigger immune responses, including calcium influx, ROS production, MAPK phosphorylation, and the expression of PTI-related genes, subsequently enhancing the resistance of tomato and pepper to P. syringae pv. tomato DC3000 and Sclerotinia sclerotiorum (40). A recent study showed that mannan oligosaccharides can serve as DAMPs to trigger multiple defense responses against *X. oryzae* and *P. nicotianae* (26). In this study, the ShAM1-degrading extracts of rice cell walls activated plant immune responses (Fig. 6), suggesting that the oligosaccharides released by ShAM1 served as DAMPs. However, which cell wall oligosaccharides (disaccharides, trisaccharides, tetrasaccharides, or other identified oligosaccharides) act as DAMPs needs to be further verified, and these oligosaccharides need to be purified. To the best of our knowledge, this is the first report about α-mannosidases that function to activate immunity via DAMP-related mechanisms.

One of the hallmark traits of probiotic treatments is low cost. For instance, beneficial bacteria induce mild but effective immune activation in the absence of pathogens (56), which leads plants in a priming stage to accelerate defense responses in the event of a challenger attack (57). A study on the costs and benefits of priming in *Arabidopsis* demonstrated that the fitness costs of priming are substantially lower than those of the directly induced defense against pathogens (58). In addition, it was shown that the benefits of priming outweigh its costs when disease occurs (59). Thus, the cost-efficient priming-inducing strategy is increasingly considered for application in disease management. To date, beneficial rhizobacteria and mycorrhizal fungi or virulent/avirulent pathogens have been reported as priming elicitors (60, 61). Some natural or synthetic compounds, such as certain agrochemicals, lipopolysaccharide, chitosan, and the flagellin flg22, were also revealed as priming-inducing agents (62, 63). However, knowledge of endophytes as priming elicitors to stimulate defense responses is still in its infancy (64). Similarly, reports about proteins playing roles in priming induction are scarce (65). This study revealed a new resource for priming-inducing agents from endophyte-derived proteins. The accumulations of H_2_O_2_ and callose in ShAM1-pretreated rice were faster and stronger than those in control rice after infection by M. oryzae, indicating that after the immune response induced by ShAM1, rice was permanently in the primed (alarm) state and efficiently protected against pathogen stress (Fig. 3).

Signaling hormones (i.e., SA, JA, and ET) are well known to play important roles in regulating plant immune responses to various microorganisms and herbivores (66). Generally, JA/ET and SA signaling are extensively reported to be associated with probiotic-plant and pathogen-plant interactions, respectively (67). In this study, the resistance to M. oryzae of ShAM1-pretreated rice mainly relied on the JA/ET pathway (Fig. 4F and G), while the expression of SA signal-related genes remained lower than that in control rice over the entire M. oryzae infection period (Fig. 4D and E). These results indicate that immune regulation in plants by active components derived from probiotics is also consistent with the mechanism of action probiotics. Similarly, the protein elicitor PemG1 from Magnaporthe grisea inhibited disease development in rice and was shown to be dependent on the SA signaling pathway but not the JA/ET pathway (68). *M. grisea* is considered a pathogenic fungus; thus, PemG1 acts as a virulence factor to induce the pathogen-related SA pathway to trigger the host immune response. However, increasing evidence demonstrates that virulence proteins related to the SA signaling pathway promote the infection of pathogens and make plants susceptible to disease (69). However, avirulence proteins involved in the JA/ET signaling pathway can interact directly or indirectly with disease resistance proteins in host plants and then induce plant defense against the pathogen (70). This result was in agreement with the observation for ShAM1. Another piece of evidence is about INF1, an elicitin from the pathogen Phytophthora infestans. INF1 is regarded as an avirulence factor because it can activate JA/ET-mediated signaling pathways and induce resistance to bacterial wilt disease in tomato (71). We subsequently verified the regulatory effect of this avirulence protein INF1 on rice blast disease and found that INF1-pretreated rice exhibited enhanced resistance to *M. grisea*, which was indeed dependent on the JA/ET but not the SA signaling pathway (Fig. S9). Taken together, the results showed that ShAM1 derived from endophytes can be used as a more environmentally friendly and safe prevention strategy for plant disease control. Moreover, the results also provide new evidence for the interaction mode of endophytic actinomycetes-plant symbionts.

## MATERIALS AND METHODS

### Strains and culture conditions.

Endophyte *S. hygroscopicus* OsiSh-2 (GenBank accession no. GCA_001705785.1; China General Microbiological Collection Center accession no. CGMCC-8716, https://cgmcc.net/.) was isolated from rice sheaths and maintained on International *Streptomyces* Program 2 (ISP2) solid medium at 30°C as described previously (72). The growth and conidiation conditions for the pathogenic fungus M. oryzae 70-15 were the same as described previously (73). Spore suspensions of OsiSh-2 and 70-15 were prepared as described previously (74).

### Preparation, purification, and identification of secreted proteins from OsiSh-2.

To produce large quantities of secreted proteins, OsiSh-2 was grown in ISP2 broth at 30°C and 170 rpm. Six to 8 days of cell-free culture filtrate (CFC) was collected and filtered through a 0.22-μm-pore Millex-GP syringe filter unit (Millipore). Subsequently, 40%, 60%, and 80% saturated proteins were precipitated overnight at 0°C by the addition of 22.6 g, 36.1 g, and 51.6 g (NH4)_2_SO_4_ per 100 mL CFC, respectively. The precipitate was collected by centrifugation at 12,000 × *g* for 30 min at 4°C and then resuspended in 20 mM Tris-HCl (pH 7.5). The solution was dialyzed (7,000-Da molecular weight cutoff) to remove ammonium sulfate and passed through a 0.22-μm filter. The resuspended proteins were loaded onto anion-exchange chromatography on a DEAE Fast Flow (FF) column (GE Healthcare) equilibrated with Tris-HCl and eluted with a NaCl gradient from 0.0 to 1.0 M in Tris-HCl and then subjected to fast protein liquid chromatography (FPLC) separation. The fractions corresponding to absorbance peaks at 280 nm were collected. The collected fractions were further desalted and concentrated using 3-kDa ultrafiltration membranes (Millipore). The selected fractions were further purified by a Superdex 200 Increase 10/300 GL column (GE Healthcare). The collected fractions were then concentrated by lyophilization. Then, select protein samples with high and no HR activity were analyzed by nano-liquid chromatography-tandem mass spectrometry (NanoLC-MS/MS) as described previously (75).

### Plasmid construction and preparation.

The tested genes were amplified from *S. hygroscopicus* OsiSh-2 DNA using Phanta Max Super-Fidelity DNA polymerase (Vazyme Biotech, Nanjing, China). All sequences of the above genes were cloned separately into the vector pCAMBIA-1300-FLAG. Then, the results were verified by DNA sequencing (Sangon Biotech, Shanghai, China). Subsequently, the recombinant plasmids were introduced into A. tumefaciens strain GV3101, and then A. tumefaciens-mediated transformation (agroinfiltration) was used to transiently express the 15 corresponding coding genes in tobacco leaves. The primers used for transient expression are listed in Table S3.

### Expression and purification of recombinant ShAM1 protein.

The purified fragment of the ShAM1 gene was cloned into the pCold TF vector and transformed into E. coli strain BL21(DE3) for expression. E. coli was grown in LB medium to an optical density at 600 nm (OD_600_) of 0.6 at 28°C. ShAM1 expression was induced by 0.5 mM isopropyl β-d-1-thiogalactopyranoside (IPTG) at 16°C overnight. Recombinant ShAM1 protein was purified using Ni-nitrilotriacetic acid (NTA) agarose (Thermo Scientific) according to the manufacturer’s instructions. The primers used for protein expression are listed in Table S3.

### HR activity assays.

The purified recombinant ShAM1 (3 μM) was sprayed onto the leaf surfaces of 2-week-old rice seedlings. Photographs were taken 48 h after inoculation. To infiltrate the purified recombinant ShAM1 into the rice leaves, the detached leaves of 2-week-old rice were lightly wounded with a mouse ear punch and then 10 μL of purified recombinant ShAM1 (3 μM) was infiltrated through the wounded areas. Rice leaves were placed in petri dishes with a light/dark light cycle of 16 h/8 h for 48 h. Photographs were taken 48 h after infiltration. Both treatments used pCold TF as control. As for the other plant species (*Arabidopsis*, pepper, tobacco, tomato, and cucumber), their leaves were infiltrated with the purified recombinant ShAM1 (3 μM) by using a syringe without a needle. pCold TF was used as control. Leaves were photographed at 3, 3, 7, 7, and 8 days postinfiltration, respectively. Three replicates were performed for each treatment.

### RNA extraction and gene expression analysis.

Rice leaves (0.05 g) were collected for total RNA extraction by using a plant total RNA isolation kit according to the manufacturer’s instructions (Sangon Biotech, Shanghai, China). Then, reverse transcription reactions were performed using 1 μg of total RNA with HiScript II Q RT SuperMix for quantitative PCR (qPCR) (+gDNA wiper) with genomic DNA (gDNA) eraser (Vazyme Biotech, Nanjing, China). Gene expression was analyzed by qRT-PCR as described previously (31). The transcript data were normalized using β-actin mRNA expression levels as the internal reference. Independent experiments were repeated twice, and all reactions were performed in triplicate. The primers used for qRT-PCR are listed in Table S3.

### H_2_O_2_ accumulation and callose deposition assays.

The rice leaves were stained with 1 mg/mL 3,3′-diaminobenzidine (DAB) (Biotopped) solution overnight at 28°C. After incubation with DAB, leaves were fixed and cleared in absolute alcohol with frequent changes of the fresh solution, and then the red-brown precipitate formed by polymerized DAB in the presence of H_2_O_2_ was examined under a stereomicroscope (MZ62+MSX1; Mshot, Guangzhou, China). Triplicate samples were performed for each treatment. For the callose deposition assay, the rice leaves were fixed and cleared in absolute alcohol with frequent changes of fresh solution, and the chlorophyll was removed. The transparent leaves were washed with 70 mM sodium phosphate buffer three times and then incubated in stain solution (70 mM sodium phosphate buffer; 0.01% aniline blue) (Macklin) for 2 h in the dark. Then, the leaves were observed using an inverted microscope under UV light (340 to 380 nm) (TS2R-FL; Nikon, Tokyo, Japan). The abundance of H_2_O_2_ accumulation and callose intensities are represented as the percentages of relative DAB and callose intensities, respectively, which were calculated by using Photoshop CS6 and ImageJ based on counting the numbers of DAB staining pixels and callose staining pixels. At least 6 typical photographs were selected from 10 randomly selected seedlings in each treatment. Three replicates were performed for each treatment.

### MAPK assays.

The MAPK assays were described previously with some modifications (16). Briefly, rice suspension cells were subcultured in fresh medium for 3 days. Aliquots of 500 mg of cells were suspended in 5 mL preincubation medium (3% sucrose and 10 mM MES [morpholineethanesulfonic acid] in 5% culture medium [pH 5.8]) and incubated under culture conditions for 4 h. Total proteins were extracted with plant radioimmunoprecipitation assay (RIPA) lysis buffer (P0045; Beyotime Biotechnology, Shanghai, China) and phosphatase inhibitor cocktail (B15001; Bimake, Shanghai, China) from rice suspension cells subjected to treatment with ShAM1 or ShAM1-digested cell wall extract. Analysis was carried out by SDS–PAGE and Western blotting using a phosphorylation-specific p38 MAPK antibody (1:5,000 dilution) (Cell Signaling).

### Rice leaf-inoculation assays.

Rice leaf-inoculation assays were performed following the method described previously, with some modifications (31). Detached leaves of 4-week-old rice were lightly wounded with a mouse ear punch, and then 10 μL of spore suspension (concentration of 5 × 10^5^ conidia/mL in 0.02% Tween 20) of M. oryzae was added to the wounded leaves. The inoculated rice leaves were placed in plates with water to restore humidity, kept in darkness overnight at 26°C, and then transferred to normal growth conditions with a 16-h/8-h light/dark photoperiod for 5 days. Photographs were taken 5 days after inoculation.

### Fungal biomass analysis.

The fungal biomass in rice plants was quantified as described previously, with slight modification (31). In brief, 0.05 g of rice tissue was collected for DNA extraction using an Ezup spin column plant genomic DNA purification kit (Sangon Biotech, Shanghai, China) according to the manufacturer’s specifications. The corresponding fungal biomass was examined by qPCR using a Bio-Rad CFX96 real-time system (Bio-Rad Laboratories, Hercules, CA, USA). The relative fungal growth was calculated using the threshold cycle value (*C_T_*) of *MoPot2* DNA against the *C_T_* of *OsUbq* DNA. All experiments were performed with three biological replicates. The primers for DNA-based qRT-PCR are listed in Table S3.

### Enzyme activity assays.

The relative activity of ShAM1 was evaluated using different substrates at a final concentration of 5 mM. For polysaccharide substrates such as LBG, microcrystalline cellulose, and xylan, the enzyme activity was measured using the 3,5-dinitrosalicylic acid (DNS) method described in our previous study (76). For *p*-nitrophenol (pNP) substrates, 150 μL of each substrate was incubated with 50 μL of recombinant ShAM1 (3 mM) in MES buffer (100 mM [pH 5.5]) at 40°C for 12 h. The reaction was terminated by adding 50 μL of 10% Na_2_CO_3_, and then the absorbance was measured at 405 nm. pNP was used as a standard. All experiments were performed in triplicate. Following previously described methods for the characterization of proteins (76), the optimal pH for ShAM1 activity was assessed in the pH range from 3 to 12, and the optimal temperature was determined over a temperature range of 20 to 65°C. For the pH stability determination, protein samples were incubated at 4°C for 24 h in buffers with different pH values from 3 to 12. The thermostability of ShAM1 at pH 5.5 was measured when the protein samples were incubated at temperatures ranging from 10 to 80°C for 30 min. The effects of metal ions (Mg^2+^, Ca^2+^, Co^2+^, Zn^2+^, Ni^2+^, Fe^2+^, Cu^2+^, Mn^2+^, Pb^2+^, Al^3+^, Fe^3+^, Na^+^, and K^+^) were studied by adding metal ions at a final concentration of 5 mM into the assay buffer.

### Rice cell wall hydrolysis assays.

For rice cell wall isolation, 4-week-old rice seedlings (10 to 20 g) were harvested, frozen immediately in liquid nitrogen, and then ground into powder. The material was extracted with 70% ethanol three times at 70°C for 1 h, washed three times with double-distilled water (ddH_2_O), and then dried at 45°C to a constant weight. Aliquots (1 mL) of a 50-mg/mL suspension of the isolated rice cell wall in 100 mM MES (pH 5.5), were incubated for 12 h at 40°C with 10 μg ShAM1 proteins. After incubation, the samples were boiled for 20 min, statically cooled to room temperature and centrifuged at 12,000 × *g* for 30 min. The supernatants were collected and filtered through a 0.22-μm-pore microporous membrane (Millipore) for analysis.

### Derivatization by O-ethyloximation (EtOx)/trimethylsilylation (TMS).

The hydrolysate of ShAM1 was derivatized before testing. Ten milliliters of the ShAM1-digested cell wall extracts was lyophilized. The lyophilized sample was dissolved in 800 μL of anhydrous pyridine containing 40 mg/mL *O*-ethylhydroxylamine hydrochloride and heated at 70°C for 1 h. Subsequently, 800 μL of a solution of 1.5 mg/mL 4-(dimethylamino) pyridine (DMAP) in pyridine was added to the mixture, followed by 800 μL of *N*,*O*-bis (trimethylsilyl) trifluoroacetamide (BSTFA) (containing 10% trimethylsilyl chloride [TMCS]). The mixture was heated at 70°C for 2 h. The derivatized samples were lyophilized and then kept at −20°C until analysis.

### GC-MS and MALDI-TOF MS analysis of oligosaccharides in hydrolyzed cell walls.

The oligosaccharides released from ShAM1-hydrolyzed cell walls were detected by GC-MS and MALDI-TOF MS. GC-MS analysis was performed on a Nexis GC-2030 (Shimadzu, Japan). An Rxi-5Sil MS column (40 mm by 0.25 mm by 0.25 μm) (Shimadzu Scientific Instruments Scientific, Folsom, CA, USA) was used. The GSMS-QP2020 NX carrier gas was helium, and the injector temperature was 280°C. The column flow rate was 0.87 mL/min. The purge flow rate was 44.1 mL/min. The oven program was as follows: from 50°C (2 min) to 280°C (20 min) at 5°C/min. The MS parameters were as follows: electron impact (EI) ionization voltage, 70 eV; source pressure, 65.8 kPa; source temperature, 230°C; and scan range, 50 to 1,000 Da. The derivatized samples were dissolved in ethyl acetate (600 μL) and filtered through a 0.22-μm-pore microporous membrane before injection. The injection volume and split ratio were 1 μL and 50:1, respectively. MALDI-TOF MS analysis was performed on a Bruker ultraflextreme MALDI-TOF/TOF mass spectrometer. Mass spectra were obtained in the positive linear and reflectron mode. The mass range was 510 to 720 Da. The filtered products of different protein-hydrolyzed rice cell walls (1 μL) were mixed with 1 μL of 2,5-dihydroxy benzoic acid solution (10 mg/mL in 2:1 acetonitrile-water containing 0.1% trifluoroacetic acid [TFA]). The mixture was then spotted on the target plate with 1 μL of 3-aminoquinoline (10 mg/mL) and dried for MALDI-TOF MS analysis.

### Statistical analysis.

Statistical significance was analyzed using appropriate statistical tests, including by one-way repeated-measures analysis of variance (ANOVA) using SPSS 17.0 software (Chicago, IL). The significant differences between the treatments were determined according to Tukey’s *post hoc* test or two-tailed Student's *t* test for evaluating the difference between two groups. Duncan’s multiple-range tests were used to compare the means for multiple groups. A *P* value of <0.05 was considered statistically significant.

### Data availability.

All data supporting the findings of this study are available within the article and the supplemental material files or are available from the corresponding author upon reasonable request.
