# Supplementary material for: The Rice Endophyte-Derived α-Mannosidase ShAM1 Degrades Host Cell Walls To Activate DAMP-Triggered Immunity against Disease
Source: Microbiol Spectr. 2023 May 8;11(3):e04824-22. doi: 10.1128/spectrum.04824-22 (PMC10269736; doi:10.1128/spectrum.04824-22)
Supplement: Supplemental file 1 — Fig. S1 to S9 and Table S1 to S3. Download spectrum.04824-22-s0001.pdf, PDF file, 1.0 MB [file spectrum.04824-22-s0001.pdf]

Supplemental Material

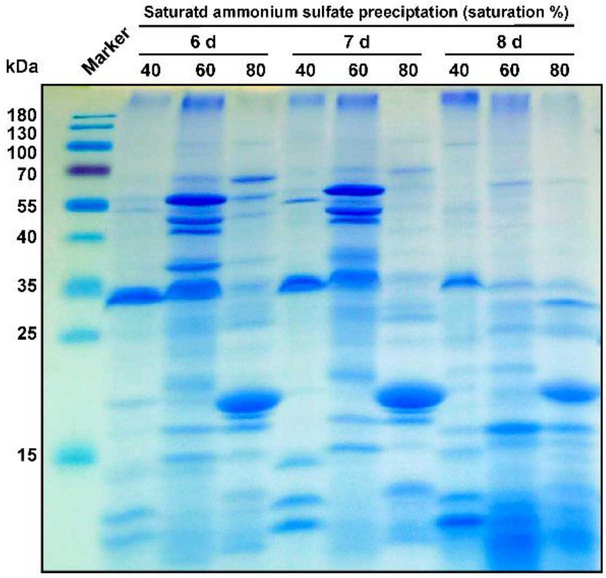

Fig S1. Different saturated ammonium sulfate concentrations were used for 6-8 days of precipitation, and the proteins were detected by Coomassie brilliant blue staining in an SDS–PAGE gel.

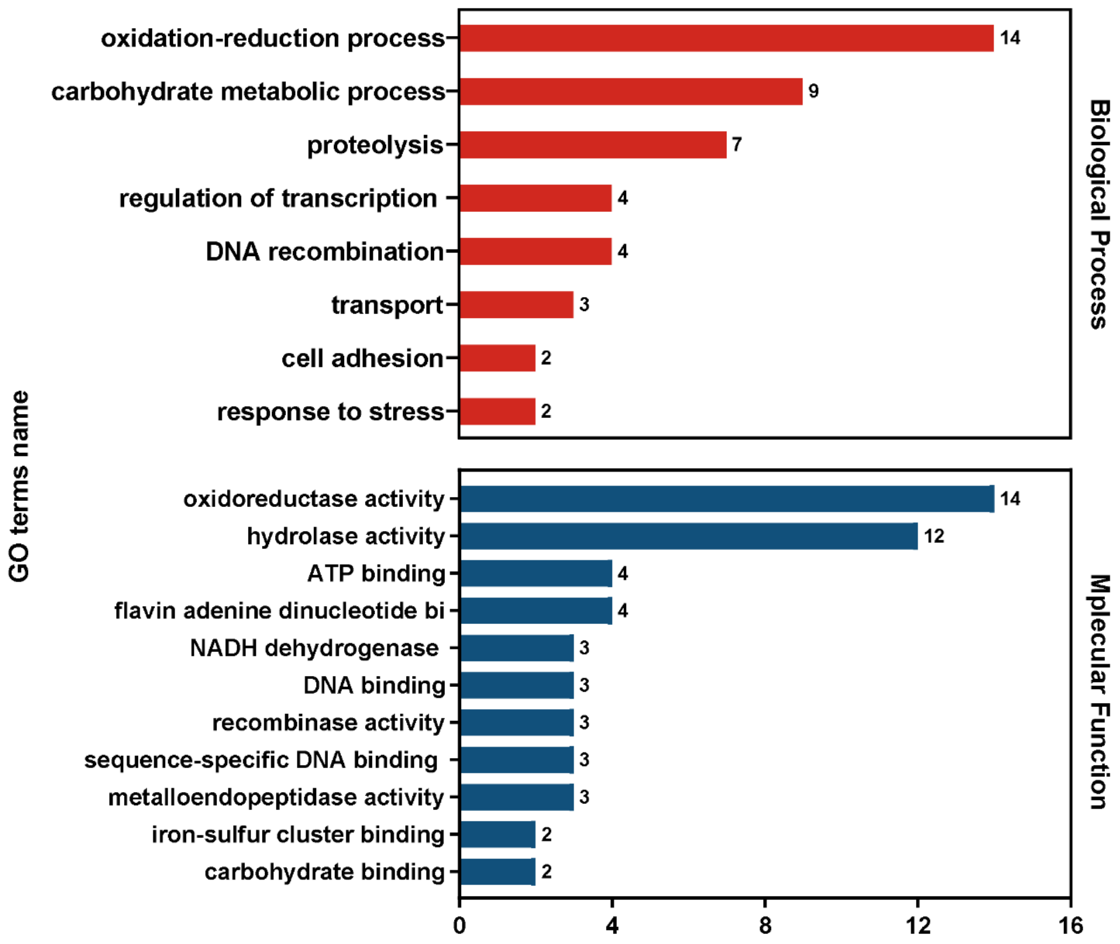

Fig S2. GO term-based enrichment analysis for the differential proteins of Peak 1 with the hypersensitive response and peak 4 without the hypersensitive response isolated by gel chromatography**.**

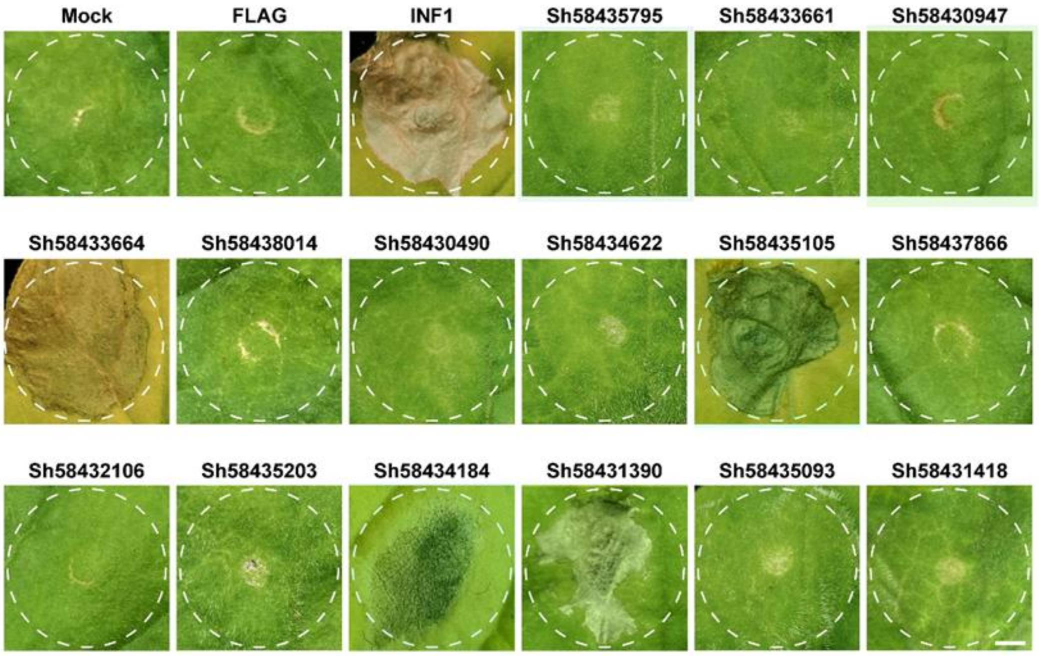

Fig S3. The identified proteins were screened for hypersensitive response activity using the tobacco transient expression system. The leaves of 4-week-old tobacco were inoculated with Agrobacterium strains carrying the indicated gene in the vector pCAMBIA-1300-FLAG. INF1 and the vector pCAMBIA-1300-FLAG were used as positive and negative controls, respectively. The Agrobacterium tumefaciens strain GV3101 was used as a mock treatment. Images were taken 7 d after inoculation. Scale bars: 0.5 cm.

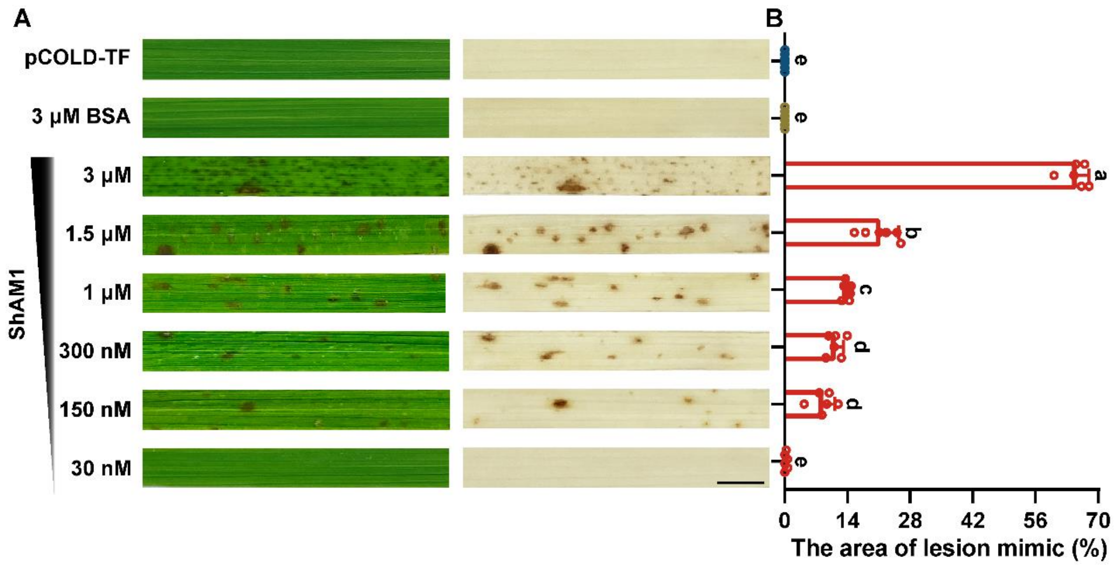

Fig S4. Recombinant ShAM1 at different concentrations induced hypersensitive response activity in rice. (A) Rice leaves sprayed with different concentrations of purified recombinant ShAM1 protein (30 nM-3 μM). Images were taken 48 h after treatment. The vector pCold TF was used as a control. Left pictures, directly photographed 48 h post-inoculation. Right pictures, photographed 48 h post-inoculation after decolorizing with ethanol. Each experiment was repeated three times with similar results. Scale bars: 1 cm. (B)The area of the lesion mimic was calculated by ImageJ software. The data shown indicate the means ± SDs. Bars with different letters are significantly different (ANOVA, $P<0.05$) according to Duncan's multiple-range test.

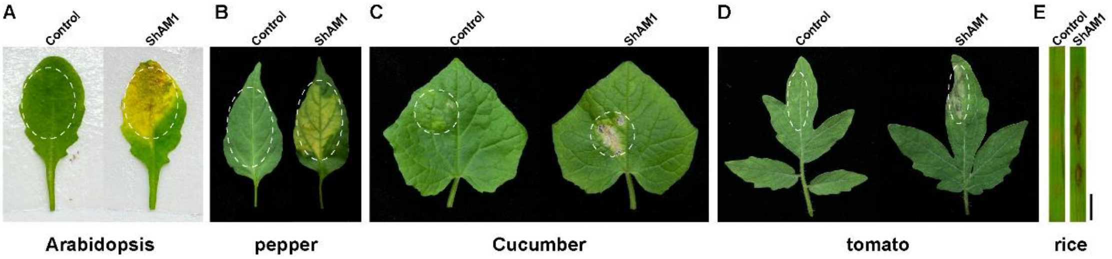

Fig S5. ShAM1 could induce HR in various plant species. HR in five other species of plants triggered by infiltrating 3 μM recombinant ShAM1. Vector pCold TF was used as the control. Representative leaves are shown of Arabidopsis (A), pepper (B), cucumber (C), tomato (D), and rice (E). Each experiment was repeated three times with similar results, Scale bars: 1 cm.

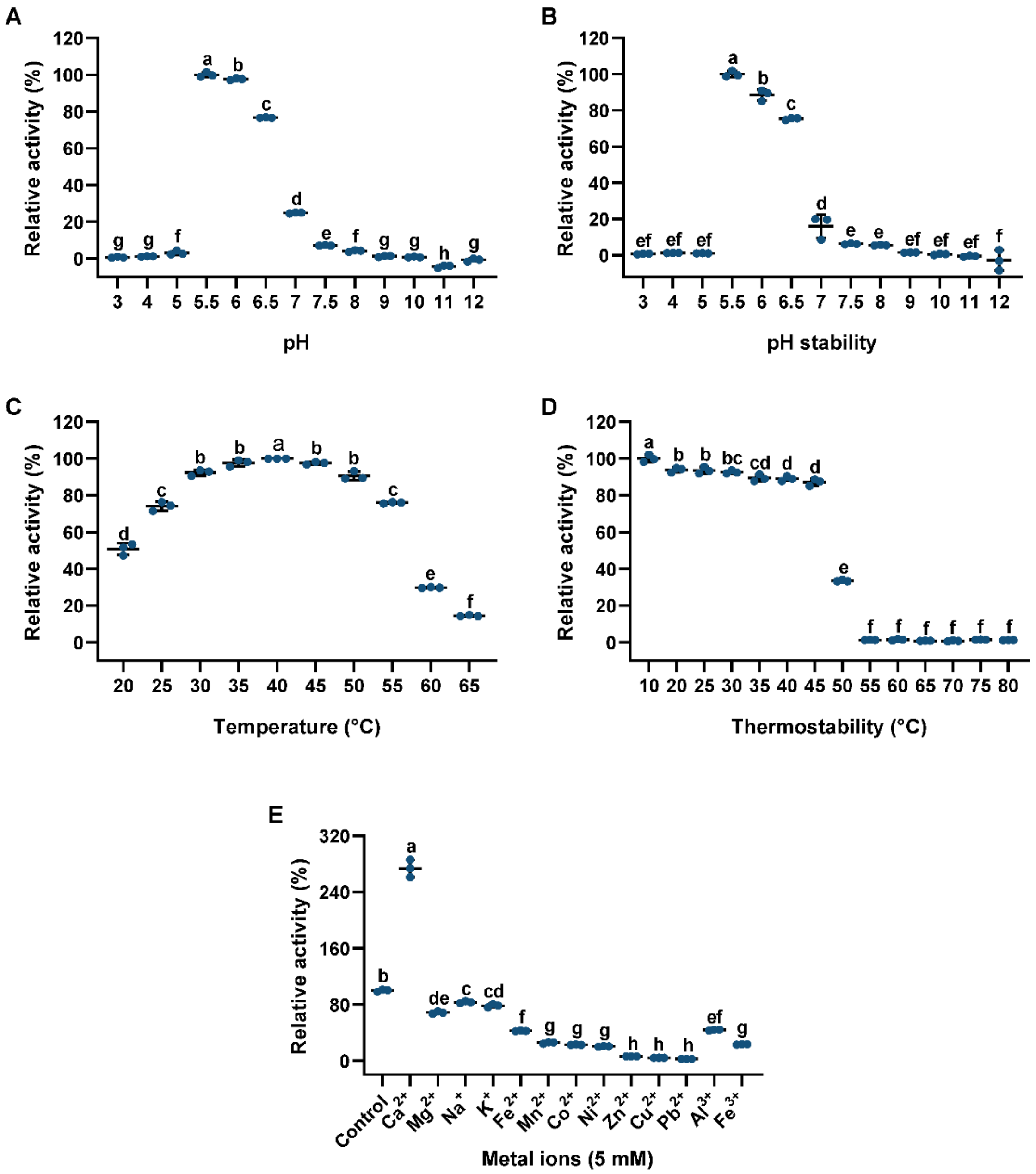

Fig S6. Biochemical properties of recombinant ShAM1. (A) The optimal pH of recombinant ShAM1 activity was 5.5. (B) The optimum pH of ShAM1 was measured at 40°C for 12 h. The pH stability of the recombinant ShAM1 protein ranged from 5.5 to 6.5. The pH was investigated at 4°C, and the residual activity was measured under standard assay conditions. (C) The optimal temperature for recombinant ShAM1 activity was 40°C. The optimum temperature of ShAM1 was measured at pH 5.5 for 12 h. (D) The thermostability of ShAM1 was analyzed at pH 5.5 for 30 min, and the residual activity was measured under standard assay conditions. (E) The activity of ShAM1 was measured in 100 mM MES buffer (pH 5.5) containing 5 mM corresponding metal ions at 40°C. The above reactions were started by the addition of pNP-α-D-man (5 mM), and then the absorbance

was measured at 405 nm. The data shown indicate the means ± SD. Bars with different letters are significantly different (ANOVA, $P<0.05$) according to Duncan's multiple-range test. The experiment was repeated three times with similar results.

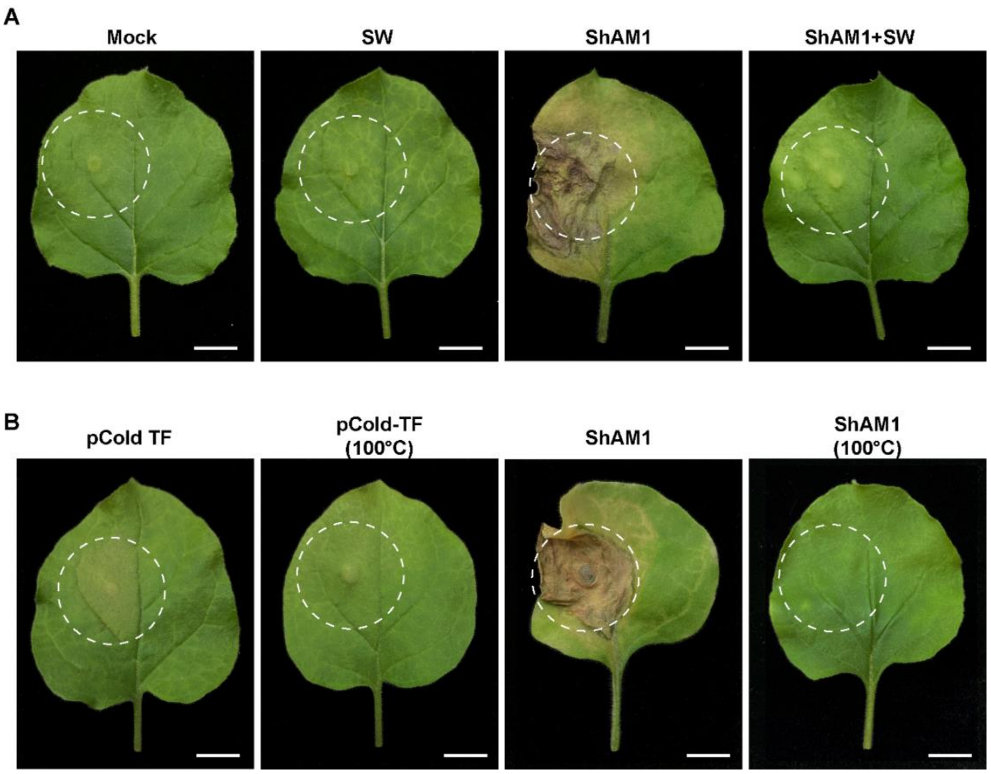

Fig S7. Swainsonine and high temperature inhibited ShAM1-induced HR in tobacco. Swainsonin (SW) inhibitor-pretreated recombinant ShAM1 for 30 min (A) or high temperature (preincubated at 100°C for 20 min) (B) and then injected into tobacco. Buffer was used as Mock. Vector pCold TF served as the control treatment. Images were taken 5 d after injection. Scale bars: 1 cm. Each experiment was repeated three times with similar results.

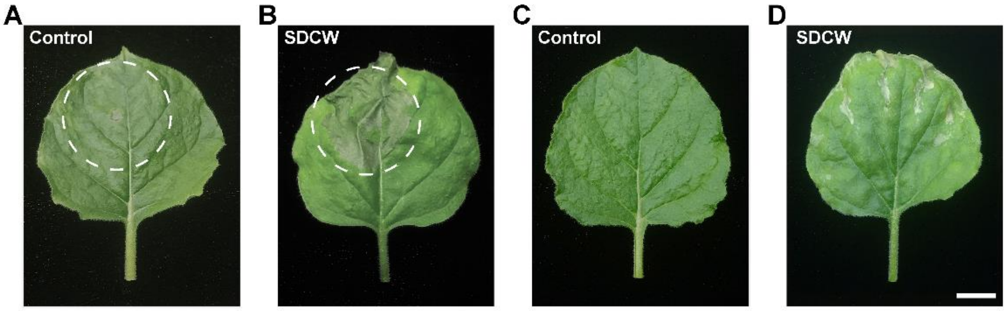

Fig S8. ShAM1-digested cell wall (SDCW) extracts induced HR in tobacco. The isolated tobacco cell walls were incubated with 10 μg of recombinant ShAM1 or pCold TF. After incubation, samples were boiled for 20 min to denature the protein. A-D. The leaves of four-week-old tobacco were injected (A-B) or sprayed (C-D) with ShAM1-digested cell wall extracts or control. pCold TF-digested cell wall extracts were used as controls. Images were taken 3 d after treatment. The experiment was repeated three times with similar results. Scale bars: 1 cm.

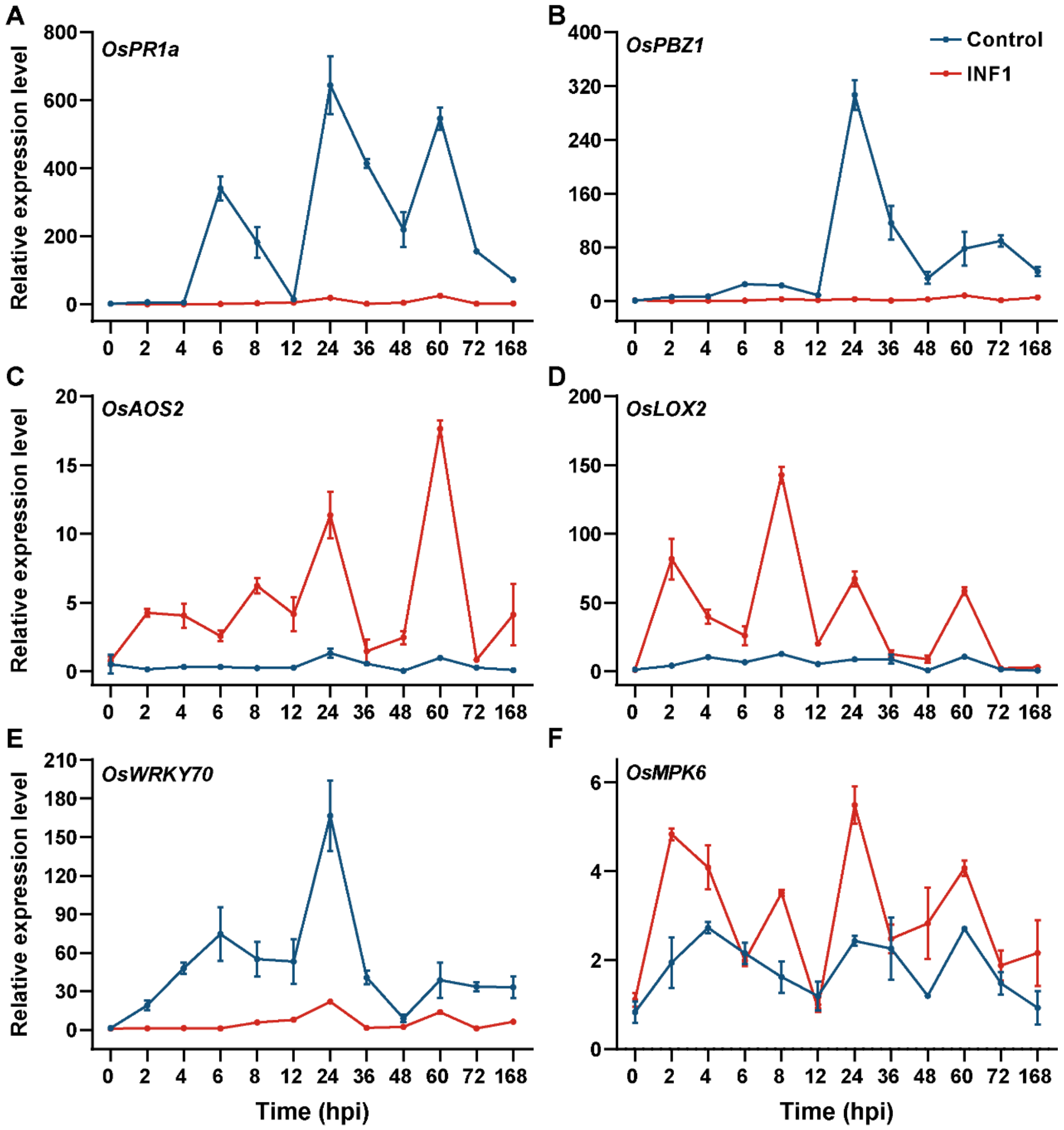

Fig S9. INF1 enhanced the expression of defense-related genes in rice after *M. oryzae* infection. A-F) The expression levels of defense-related genes were significantly upregulated in INF1-pretreated rice after *M. oryzae* infection. INF1 was inoculated with 2-week-old rice seedlings and then infected with *M. oryzae* after recovery for seven days, and samples were collected for qRT–PCR at 5 days postinfection by *M. oryzae* in rice leaves. The data shown indicate the means ± SDs (n = 3, n refers to technical replicates). Each experiment was repeated three times with similar results.

Table S1. Differential proteins of Peak 1 with the hypersensitive response and peak 4 without the hypersensitive response identified by NanoLC/MS-MS

Classify	Function	Gene ID	Description	Sequence coverage [%]	Score	Unique Peptides	MW [kDa]
Hydrolases enzymes	Glycoside hydrolases	58435795	alpha-N-acetylglucosaminidase	18.68	9.54	4	113.6
		58433661	alpha-1,2-mannosidase	18.66	1.88	1	136.8
		58430947	beta-mannosidase	24.38	38.56	9	71.4
		58433664	alpha-mannosidase	11.81	2.57	2	113.7
		58438014	alpha-galactosidase	10.47	1.93	1	78.0
		58430490	beta-galactosidase	16.27	1.89	1	70.4
		58434622	glycoside hydrolase	20.99	3.91	1	28.2
		58435105	glycosyl hydrolase	16.46	24.03	6	85.9
	Others	58437866	hydrolase	7.49	2.22	1	32.9
		58432106	hydrolase	7.61	2.21	1	38.0
		58435203	hydrolase	21.03	2.05	1	24.6
		58434184	alpha/beta hydrolase	14.58	2.00	2	32.2
		58431390	alpha/beta hydrolase	22.57	9.34	2	49.6
		58435093	Serine hydrolase	25.37	10.62	3	43.6
		58431418	hydrolase	30.11	20.72	9	56.9

Table S2. Substrate specificity of recombinant ShAM1.

Enzyme	Substrates	Relative activity (%)
α-mannosidase	4-Nitrophenyl-α-D-mannopyranoside	100
β-mannosidase	4-Nitrophenyl-β-D-mannopyranoside	nd
α-glucosidase	4-Nitrophenyl-α-D-glucopyranoside	nd
β-glucosidase	4-Nitrophenyl-β-D-glucopyranoside	nd
β-mannosidase	locust bean gum	nd
cellulase	microcrystalline cellulose	nd
xylanase	xylan	nd

nd: not detectable.

Table S3. Primers used for this study

Prime name	Primer sequence (5'-3')	Purpose
pCAMBIA1300-FLAG-Sh58435795-F	CTGAGCGGTACCCGGGGATCCATGAGCGAC	Transient expression in tobacco
pCAMBIA1300-FLAG-Sh58435795-R	TTGGTCGACTCTAGAGGATCCCTACGGCGT	Transient expression in tobacco
pCAMBIA1300-FLAG-Sh58433661-F	CTGAGCGGTACCCGGGGATCCGTGGCGGCC	Transient expression in tobacco
pCAMBIA1300-FLAG-Sh58433661-R	TTGGTCGACTCTAGAGGATCCTCAGCTCAG	Transient expression in tobacco
pCAMBIA1300-FLAG-Sh58430947-F	CTGAGCGGTACCCGGGGATCCATGCGACGC	Transient expression in tobacco
pCAMBIA1300-FLAG-Sh58430947-R	TTGGTCGACTCTAGAGGATCCTCATGTCAG	Transient expression in tobacco
pCAMBIA1300-FLAG-Sh58433664-F	CTGAGCGGTACCCGGGGATCCATGCCCTCAAG	Transient expression in tobacco
pCAMBIA1300-FLAG-Sh58433664-R	TTGGTCGACTCTAGAGGATCCTCAGCCGCG	Transient expression in tobacco
pCAMBIA1300-FLAG-Sh58438014-F	CTGAGCGGTACCCGGGGATCCGTGGTCCAT	Transient expression in tobacco
pCAMBIA1300-FLAG-Sh58438014-R	TTGGTCGACTCTAGAGGATCCCTACACGCG	Transient expression in tobacco
pCAMBIA1300-FLAG-Sh58430490-F	CTGAGCGGTACCCGGGGATCCATGACGCAC	Transient expression in tobacco
pCAMBIA1300-FLAG-Sh58430490-R	TTGGTCGACTCTAGAGGATCCTCATCGCCC	Transient expression in tobacco

pCAMBIA1300-FLAG-Sh58434622-F	CTGAGCGGTACCCGGGGATCCGTGGCCCTG	Transient expression in tobacco
pCAMBIA1300-FLAG-Sh58434622-R	TTGGTCGACTCTAGAGGATCCTCAGGACGTGCG	Transient expression in tobacco
pCAMBIA1300-FLAG-Sh58435105-F	CTGAGCGGTACCCGGGGATCCGTGCACAGG	Transient expression in tobacco
pCAMBIA1300-FLAG-Sh58435105-R	TTGGTCGACTCTAGAGGATCCTCAGCCGGT	Transient expression in tobacco
pCAMBIA1300-FLAG-Sh58437866-F	CTGAGCGGTACCCGGGGATCCGTGACCGGATTC	Transient expression in tobacco
pCAMBIA1300-FLAG-Sh58437866-R	TTGGTCGACTCTAGAGGATCCTCAGGCCAG	Transient expression in tobacco
pCAMBIA1300-FLAG-Sh58432106-F	CTGAGCGGTACCCGGGGATCCATGCGTAAG	Transient expression in tobacco
pCAMBIA1300-FLAG-Sh58432106-R	TTGGTCGACTCTAGAGGATCCCTACTTTCC	Transient expression in tobacco
pCAMBIA1300-FLAG-Sh58435203-F	CTGAGCGGTACCCGGGGATCCATGACCAGC	Transient expression in tobacco
pCAMBIA1300-FLAG-Sh58435203-R	TTGGTCGACTCTAGAGGATCCTCAGTGCAT	Transient expression in tobacco
pCAMBIA1300-FLAG-Sh58434184-F	CTGAGCGGTACCCGGGGATCCATGAGTGAT	Transient expression in tobacco
pCAMBIA1300-FLAG-Sh58434184-R	TTGGTCGACTCTAGAGGATCCCTATGCCTTGAG	Transient expression in tobacco
pCAMBIA1300-FLAG-Sh58431390-F	CTGAGCGGTACCCGGGGATCCATGCAGCAG	Transient expression in tobacco
pCAMBIA1300-FLAG-Sh58431390-R	TTGGTCGACTCTAGAGGATCCTCAGCCCCG	Transient expression in tobacco

pCAMBIA1300-FLAG-Sh58435093-F	CTGAGCGGTACCCGGGGATCCGTGGCCACC	Transient expression in tobacco
pCAMBIA1300-FLAG-Sh58435093-R	TTGGTCGACTCTAGAGGATCCTCATCGCGC	Transient expression in tobacco
pCAMBIA1300-FLAG-Sh58431418-F	CTGAGCGGTACCCGGGGATCCGTGCTCGCC	Transient expression in tobacco
pCAMBIA1300-FLAG-Sh58431418-R	TTGGTCGACTCTAGAGGATCCTCAGAAACG	Transient expression in tobacco
pColdTF-Sh58435795-F	CTCGGTACCCTCGAGGGATCCATGAGCGAC	Protein expression
pColdTF-Sh58435795-R	GACAAGCTTGAATTCGGATCCCTACGGCGT	Protein expression
pColdTF-Sh58433661-F	CTCGGTACCCTCGAGGGATCCGTGGCGGCC	Protein expression
pColdTF-Sh58433661-R	GACAAGCTTGAATTCGGATCCTCAGCTCAG	Protein expression
pColdTF-Sh58430947-F	CTCGGTACCCTCGAGGGATCCATGCGACGC	Protein expression
pColdTF-Sh58430947-R	GACAAGCTTGAATTCGGATCCTCATGTCAG	Protein expression
pColdTF-Sh58433664-F	CTCGGTACCCTCGAGGGATCCATGCCCTCAAG	Protein expression
pColdTF-Sh58433664-R	GACAAGCTTGAATTCGGATCCTCAGCCGCG	Protein expression
pColdTF-Sh58438014-F	CTCGGTACCCTCGAGGGATCCGTGGTCCAT	Protein expression
pColdTF-Sh58438014-R	GACAAGCTTGAATTCGGATCCCTACACGCG	Protein expression
pColdTF-Sh58430490-F	CTCGGTACCCTCGAGGGATCCATGACGCAC	Protein expression

pColdTF-Sh58430490-R	GACAAGCTTGAATTCGGATCCTCATCGCCC	Protein expression
pColdTF-Sh58434622-F	CTCGGTACCCTCGAGGGATCCGTGGCCCTG	Protein expression
pColdTF-Sh58434622-R	GACAAGCTTGAATTCGGATCCTCAGGACGTGCG	Protein expression
pColdTF-Sh58435105-F	CTCGGTACCCTCGAGGGATCCGTGCACAGG	Protein expression
pColdTF-Sh58435105-R	GACAAGCTTGAATTCGGATCCTCAGCCGGT	Protein expression
pColdTF-Sh58437866-F	CTCGGTACCCTCGAGGGATCCGTGACCGGATTC	Protein expression
pColdTF-Sh58437866-R	GACAAGCTTGAATTCGGATCCTCAGGCCAG	Protein expression
pColdTF-Sh58432106-F	CTCGGTACCCTCGAGGGATCCATGCGTAAG	Protein expression
pColdTF-Sh58432106-R	GACAAGCTTGAATTCGGATCCCTACTTTCC	Protein expression
pColdTF-Sh58435203-F	CTCGGTACCCTCGAGGGATCCATGACCAGC	Protein expression
pColdTF-Sh58435203-R	GACAAGCTTGAATTCGGATCCTCAGTGCAT	Protein expression
pColdTF-Sh58434184-F	CTCGGTACCCTCGAGGGATCCATGAGTGAT	Protein expression
pColdTF-Sh58434184-R	GACAAGCTTGAATTCGGATCCCTATGCCTTGAG	Protein expression
pColdTF-Sh58431390-F	CTCGGTACCCTCGAGGGATCCATGCAGCAG	Protein expression
pColdTF-Sh58431390-R	GACAAGCTTGAATTCGGATCCTCAGCCCCG	Protein expression

pColdTF-Sh58435093-F	CTCGGTACCCTCGAGGGATCCGTGGCCACC	Protein expression
pColdTF-Sh58435093-R	GACAAGCTTGAATTCGGATCCTCATCGCGC	Protein expression
pColdTF-Sh58431418-F	CTCGGTACCCTCGAGGGATCCGTGCTCGCC	Protein expression
pColdTF-Sh58431418-R	GACAAGCTTGAATTCGGATCCTCAGAAACG	Protein expression
pColdTF-INF1-F	CTCGGTACCCTCGAGGGATCCATGAACTTT	Protein expression
pColdTF-INF1-R	GACAAGCTTGAATTCGGATCCTAGCGACGC	Protein expression
OsAOS2-F	TACCAGCCGTGCGCCACCAG	qRT-PCR
OsAOS2-R	AGGACGGAGCTGGTTGAGTGG	qRT-PCR
OsLOX2-F	AGATGAGGCGCGTGATGAC	qRT-PCR
OsLOX2-R	CATGGAAGTCGAGCATGAACA	qRT-PCR
OsWRKY70-F	CCGCTGCTGTTTTGATCATCT	qRT-PCR
OsWRKY70-R	GGAGCTAAGCTAACTCACTCCACA	qRT-PCR
OsMPK6-F	CGCACGCTCAGGGAGATC	qRT-PCR
OsMPK6-R	GGTATGATATCCCTTATGGCAACAA	qRT-PCR
OsPR1a-F	TCGTATGCTATGCTACGTGTTT	qRT-PCR

OsPR1a-R	CACTAAGCAAATACGGCTGACA	qRT-PCR
OsPBZ1-F	GTGGGAAGCACATACAAGACC	qRT-PCR
OsPBZ1-R	AGGGTGAGCGACGAGGTAG	qRT-PCR
OsActin-F	GAGTATGATGAGTCGGGTCCAG	qRT-PCR
OsActin-R	ACACCAACAATCCCAAACAGAG	qRT-PCR
MoPot2-F	ACGACCCGTCTTTACTTATTTGG	qRT-PCR
MoPot2-R	AAGTAGCGTTGGTTTTGTTGGAT	qRT-PCR
